# Neonatal Brain Injury Triggers Niche-Specific Changes to Cellular Biogeography

**DOI:** 10.1523/ENEURO.0224-24.2024

**Published:** 2024-12-19

**Authors:** Nareh Tahmasian, Min Yi Feng, Keon Arbabi, Bianca Rusu, Wuxinhao Cao, Bharti Kukreja, Asael Lubotzky, Michael Wainberg, Shreejoy J. Tripathy, Brian T. Kalish

**Affiliations:** ^1^Program in Neuroscience and Mental Health, SickKids Research Institute, Toronto, Ontario M5G 1L7, Canada; ^2^Department of Laboratory Medicine and Pathology, University of Toronto, Toronto, Ontario M5S 1A8, Canada; ^3^Department of Biological Sciences, Sunnybrook Research Institute, Toronto, Ontario M4N 3M5, Canada; ^4^Department of Molecular Genetics, University of Toronto, Toronto, Ontario M5G 1A8, Canada; ^5^Institute of Medical Science, University of Toronto, Toronto, Ontario M5G 1A8, Canada; ^6^Krembil Centre for Neuroinformatics, Centre for Addiction and Mental Health, Toronto, Ontario M5T 1R8, Canada; ^7^Division of Neurology, Department of Paediatrics, Hospital for Sick Children, Toronto, Ontario M5G 1L7, Canada; ^8^Department of Psychiatry, University of Toronto, Toronto, Ontario M5G 1A8, Canada; ^9^Prosserman Centre for Population Health Research, Lunenfeld-Tanenbaum Research Institute, Sinai Health, Toronto, Ontario M5G 1X5, Canada; ^10^Department of Physiology, University of Toronto, Toronto, Ontario M5G 1A8, Canada; ^11^Division of Neonatology, Department of Paediatrics, Hospital for Sick Children, Toronto, Ontario M5G 1L7, Canada

**Keywords:** hypoxia, neurogenesis, remyelination, spatial transcriptomics, white matter injury

## Abstract

Preterm infants are at risk for brain injury and neurodevelopmental impairment due, in part, to white matter injury following chronic hypoxia exposure. However, the precise molecular mechanisms by which neonatal hypoxia disrupts early neurodevelopment are poorly understood. Here, we constructed a brain-wide map of the regenerative response to newborn brain injury using high-resolution imaging-based spatial transcriptomics to analyze over 800,000 cells in a mouse model of chronic neonatal hypoxia. Additionally, we developed a new method for inferring condition-associated differences in cell type spatial proximity, enabling the identification of niche-specific changes in cellular architecture. We observed hypoxia-associated changes in region-specific cell states, cell type composition, and spatial organization. Importantly, our analysis revealed mechanisms underlying reparative neurogenesis and gliogenesis, while also nominating pathways that may impede circuit rewiring following neonatal hypoxia. Altogether, our work provides a comprehensive description of the molecular response to newborn brain injury.

## Significance Statement

Children born prematurely are at risk for white matter injury and cerebral dysmaturation, which predispose them to lifelong neurological impairments. Here, we used a mouse model of chronic neonatal hypoxia that mimics some of the features of preterm brain injury and performed high-resolution spatial transcriptomics using multiplexed error-robust fluorescence in situ hybridization (MERFISH). We developed a new approach to map cell–cell relationships, which revealed profound changes to the cellular organization in response to newborn brain injury. We defined cellular communication networks and signaling pathways that likely contribute to hypoxia-responsive neurogenesis and gliogenesis, as well as cell- and region-specific factors that may disrupt neurologic recovery and repair.

## Introduction

Preterm birth is the leading cause of neonatal morbidity and mortality in developed countries and affects approximately 15 million infants annually (Blencowe et al., 2013a). While significant strides have been made in neonatal health care leading to increased survival rates, the long-term impact of premature birth on the trajectory of neurodevelopment remains a major unsolved challenge. Survivors of preterm birth are at high risk for neurodevelopmental impairment, including cognitive and motor disability, as well as autism spectrum disorder, epilepsy, and attention-deficit/hyperactivity disorder (Blencowe et al., 2013b; Hirvonen et al., 2017; Rommel et al., 2017; Crump et al., 2021). The causes of brain injury in preterm infants are complex and multifactorial, but chronic exposure to hypoxia (HX) due to lung immaturity is an important driver of brain injury and maldevelopment. Importantly, white matter injury (WMI) is most prevalent in infants born at early gestational ages preceding the onset of myelination. This suggests a developmental susceptibility of immature oligodendrocyte (OL) lineage cells to HX–ischemia, oxidative stress, and impaired cerebral perfusion, leading to maturational failure (Salmaso et al., 2014). Despite this understanding, we lack therapies to protect the preterm brain or to promote recovery during this critical developmental window.

A mouse model of chronic sublethal HX, in which neonatal mice are exposed to 1 week of 10% oxygen, replicates many structural and pathophysiological aspects of human preterm injury and has been widely used to study preterm WMI (Fagel et al., 2006; Salmaso et al., 2014; Forbes et al., 2020; Jablonska et al., 2022). However, most studies have focused on injury and repair within the OL lineage, rather than brain-wide changes in the response to neonatal HX and the role of cell–cell communication networks. Importantly, there is growing recognition that the local microenvironment in specific brain regions dictates many aspects of cell fate and state (Tan et al., 2020; Herrero-Navarro et al., 2021; Joglekar et al., 2021), which highlights the necessity of characterizing brain region-specific cellular programs that govern recovery and repair. Glial identity varies based on cortical location (Spitzer et al., 2019; Hilscher et al., 2022; Stogsdill et al., 2022), and the local spatial niche may inform injury response (Hammond et al., 2019; Brandi et al., 2022). It is therefore likely, but incompletely understood, that a similar region-specific injury response occurs in the context of chronic neonatal HX.

To address this gap in knowledge, we applied spatially resolved single-cell transcriptomics [multiplexed error-robust fluorescence in situ hybridization (MERFISH)] to explore how neonatal HX affects the brain's spatial organization and signaling landscape. Unlike traditional droplet-based single-cell genomics, MERFISH captures subcellular localization of gene expression within the native tissue context, offering insight into cellular organization and architecture (Zhang et al., 2021, 2023; Allen et al., 2023). We performed MERFISH on the mouse brain following chronic sublethal neonatal HX and on age-matched normal oxygen (normoxic, NX) controls. At baseline (under NX conditions), we identified brain region-specific cell states and signaling-associated gene expression profiles in non-neuronal cells. Following neonatal HX, we identified increased oligodendrocyte precursor cell (OPC) proliferation but arrested differentiation of these cells into mature OL in the subventricular zone (SVZ) and corpus callosum. We characterized region-specific and cell type–specific gene expression changes that may inhibit functional myelination and repair, including upregulation of extracellular matrix components and other mediators known to inhibit oligodendrogenesis. Furthermore, to quantify compositional changes to the cellular microenvironment after HX, we developed a novel approach to infer changes in the spatial proximity of neighboring cell types. Using this method, we found extensive reorganization of local cellular architecture in the cortex, SVZ, and corpus callosum following HX. Overall, these results provide novel insights into the mechanisms underlying white matter regeneration in the neonatal brain and underscore the complex multicellular processes in the regenerative response to HX-induced preterm WMI.

## Materials and Methods

### Mouse model of chronic sublethal hypoxia

All animal experiments were performed in accordance with the SickKids Research Institute policies. CD-1 mice (strain 022) were purchased from Charles River Laboratories. The animals were kept in 12/12 h light/dark cycles with free access to food and chow and bred at the SickKids Research Institute. Offspring CD-1 mice were exposed to an oxygen concentration of either 10% (HX) or 21% (NX) in a HX chamber from postnatal day 3 (P3) to P11, lasting 8 consecutive days (Fagel et al., 2006; Sathyanesan et al., 2018; Jablonska et al., 2022). Mouse dams were rotated daily. At P11, mice were removed from the chamber and transferred to a room with normoxic conditions (21% oxygen) until euthanasia by CO_2_ at P21. Fresh brain tissue was harvested and immediately placed in optimal cutting temperature solution and frozen at −80°C until MERFISH processing. Male offspring were used for MERFISH experiments.

### MERFISH gene panel design

We created a panel of 497 genes (Extended Data Table 1-1) to study the cellular architecture and signaling landscape of the murine forebrain using MERFISH. To guide the identification of diverse cell types in the brain, the panel included 181 genes that are known cell type–specific markers or have been previously shown to have enriched expression in a specific population of cells. This list targeted known cell types within major forebrain structures including the cortex, white matter tracts, striatum, and SVZ. The remaining 316 genes were components of biological pathways shown previously or hypothesized to play a role in the pathophysiology of chronic neonatal HX. This included components of signaling pathways known to regulate oligodendrogenesis and myelination or that have previously been implicated in the pathophysiology of WMI such as WNT signaling (Varela-Nallar et al., 2014; Chavali et al., 2020), BMP signaling (Dizon et al., 2011; Wu et al., 2012), SHH signaling (Wu et al., 2012; Bergles and Richardson, 2015), FGF signaling (Furusho et al., 2012, 2015), EGF signaling (Aguirre et al., 2007), Ephrin signaling (Harboe et al., 2018; Y. Li et al., 2022), cytokine signaling (Kirby et al., 2019), senescence (Safaiyan et al., 2016; Nicaise et al., 2019), and oncogenes (Courtois-Cox et al., 2008). Each gene was assigned a 20-bit binary barcode which was used to decode sequential imaging data to assign detected transcripts to the appropriate gene.

### MERFISH tissue processing and sample preparation

All MERFISH sample preparation was performed under RNase-free conditions. Frozen embedded brains were cryosectioned at 10 μm thickness at −21°C and mounted onto room temperature (RT) MERSCOPE beaded coverslips (Vizgen, catalog #10500001). After adhering, sections were allowed to refreeze for 5–15 min and then fixed in 4% paraformaldehyde (PFA) diluted in 1× PBS for 15 min. Sections were washed three times with 1× PBS for 5 min each and then stored in 70% ethanol (EtOH) overnight at 4°C to permeabilize the tissue. Sections were stored in 70% EtOH for a maximum of 3 weeks.

Sample preparation was performed using the sample preparation kit (Vizgen, catalog #10400012) and the manufacturer’s instructions for unfixed tissue. First, sections were washed with 1× PBS followed by Sample Prep Wash Buffer (Vizgen, PN 20300001). Sections were incubated in Formamide Wash Buffer (Vizgen, PN 20300002) for 30 min at 37°C and then incubated in the gene panel mix for 42–46 h at 37°C. Sections were then incubated two times in Formamide Wash Buffer for 30 min each at 47°C and washed with sample prep wash buffer for at least 2 min. Sections were coated in gel embedding solution [0.05% w/v ammonium persulfate, 0.05% v/v *N*,*N*,*N*′,*N*′-tetramethylethylenediamine in Gel Embedding Premix (Vizgen, PN 20300004)] and incubated at room temperature (RT) for 1.5 h, cleared in 1:100 proteinase K in Clearing Premix (Vizgen, PN 20300003) at 37°C overnight or for a maximum of 7 d to clear lipids and proteins that may contribute to autofluorescence background noise. Prior to imaging, sections were washed two times with Sample Prep Wash Buffer, incubated in DAPI and PolyT Staining Reagent (Vizgen, PN 20300021) for 15 min on a rocking platform, incubated in Formamide Wash Buffer for 10 min, and washed again with Sample Prep Wash Buffer.

### MERFISH imaging

Imaging was performed on the MERSCOPE platform (Vizgen, catalog #10000001) according to the manufacturer’s instructions. Briefly, samples were loaded into the flow chamber of the instrument, and the desired region for imaging was selected using a low-resolution mosaic of DAPI and PolyT stains. Samples were imaged at high-resolution with a seven-plane *z*-stack and 1.5 μm spacing between adjacent *z*-planes to capture the entire 10 μm thickness of the tissue sections. Samples were then automatically imaged according to MERSCOPE imaging presets.

### MERFISH bioinformatics workflow

After imaging, transcript barcodes were decoded and assigned to the appropriate gene. MERFISH images were segmented using the Vizgen postprocessing tool (VPT) and Cellpose 2.0 (Pachitariu and Stringer, 2022), a machine learning algorithm (RRID:SCR_021716). DAPI and PolyT signals were used to delineate cell boundaries for each field of view. Individual RNA molecules were assigned to a cell based on whether they were positioned within the marked boundary. Anatomical segmentation was performed based on tissue morphology and region-specific gene expression patterns. This revealed eight gross anatomical regions: cortex (marked by high expression of *Slc7a6* and *Slc17a7*); corpus callosum, anterior commissure, and septal white matter tracts (all marked by high expression of *Cnp* and *Plp1*); SVZ (marked by high expression of *Foxj1*, *Sox2*, and *Ascl1*); caudoputamen (marked by high expression of *Foxo1*, *Crym*, and *Gad1/2*); lateral septal nucleus (marked by expression of *Sp8* and *Sp9*); bed nucleus of the stria terminalis (marked by *Cartpt*); and the regions ventral to the caudoputamen and anterior commissure, which were collectively labeled lower gray matter (high expression of *Gad1*, and *Dlx1/2*). Meninges (high expression of *Col1a1* and *Gfap*) were grouped with the cortex. The cortex was further segmented into upper, superficial cortex layers (marked by high expression of *Cux2*, *Cartpt*, *Kitl*, and *Rgs8*) and deeper cortex layers (marked by high expression of *Bcl11b*, *Fezf2*, *Otx1*, and *Tle4*).

To obtain a cell-by-gene matrix of each biological sample tissue replicate, we adapted an existing bioinformatic pipeline (Allen et al., 2023) as follows. (1) To remove segmentation artifacts of extremely small or large cells, we removed cells with a volume <50 µm^3^ or larger than three times the median volume of all cells. (2) Cells with zero RNA molecules were removed. (3) In a 10-µm^2^-thick tissue slice, the spatial position of some cells within the section resulted in partial imaging of their soma. To account for potential RNA discrepancies, gene expression for each cell was normalized by their physical volume and multiplied by 1,000. (4) Cells with total RNA counts falling below the 2% quantile or exceeding the 98% quantile were excluded. (5) Potential doublets were removed using Scrublet (RRID:SCR_018098), a program that generates artificial doublets by comparing gene expression profiles of randomly selected cells in the dataset and using a *k*-nearest neighbor to output a predicted doublet score. Cells with a Scrublet-based doublet score of greater than 0.20 were excluded from further analysis, accounting for 2.5–3.5% of cells across samples.

Data were then processed using the Seurat V5 package in R (RRID:SCR_016341). First, each dataset was normalized using Seurat's SCTransform function, and cells from each gross anatomical region were grouped into separate Seurat objects. We focused subsequent analyses on cells contained in 3 brain regions: cortex, corpus callosum, and SVZ. We integrated and scaled gene expression from cells from each of six samples (*n *= 3 HX and *n *= 3 NX). Only genes with sufficient expression across cells in that region (ranging from 475 to 495 genes) were integrated and used to compute principal components and uniform manifold approximation and projection (UMAP). Cell clustering was performed using shared nearest neighbors-cliq (SNN-cliq). Cell typing and subtyping were performed using canonical markers. To improve our cell type identification, we removed (1) clusters that did not express cell type–specific marker genes, and thus could not be assigned to a cell type, and (2) clusters of cells that expressed two or more mutually exclusive cell type–specific marker genes. The former excluded potential noncell artifacts or cells that could not be identified with the implemented gene panel, and the latter excluded potential doublets.

### Cell proportion analysis

To determine differences in cell type abundance in response to HX, we calculated cell type proportions for each cell subtype class within each region. For each biological replicate and each region, the total number of cells in each cell subtype was divided by either the total number of cells in the region or the total number of cells in the region corresponding to a broad cell class (e.g., neurons, glia, and OL lineage). Statistical differences in cell proportions were calculated using an unpaired, two-tailed, Student's *t* test comparing HX and NX (*n *= 3 each) for each cell subtype of each region.

### Cell-level proximity analysis

To understand how the composition of cellular neighborhoods is affected by neonatal HX, we developed a novel method to quantify condition-related differences in local cell type proximity. For each cell *i*, a *k*-dimensional tree was used to count the number of neighboring type *B* cells, *N*_B_, and the total number of neighboring cells (irrespective of type), *N*_total_, within a specified radius *r*. For each sample and brain region, we defined a minimum distance unit, *d*, as the median of the smallest distances between all cell pairs for each sample. A restricted radius (*r *= 5 * *d*) was selected to focus on the immediate proximity interactions between cell types. The proportion of type B cells within this radius is given by:

$$B_{\mathrm{rati}o_{i}}=\frac{N_{B}}{N_{\mathrm{total}}},$$

The *B*_ratio_ provides a normalized measure of the local proportion or density of cell type B in the proximity to a reference cell *i*. We note that *B*_ratio_ is asymmetric (e.g., the proportion of astrocytes in the proximity of neurons is different from the proportion of neurons in the proximity of astrocytes), which allows for discriminating when the neighborhood of one cell type is enriched or depleted for another cell type, but not vice versa. We further note that this analysis of local cell proximity or density is conceptually similar to the analysis of global cell proportion differences defined above.

To identify differences in local cell type proximity under NX and HX conditions, we used a generalized linear mixed-effects model implemented via the glmmTMB R package and goodness-of-fit calculations evaluated by the DHARMa package (RRID:SCR_022136). This statistical model addresses the nonindependence of within-tissue sample observations and is applied to the log-transformed *B*_ratio_, with a small offset to accommodate zeroes. This approach quantifies the impact of condition on cell type distributions, while accounting for intersample variability:

$$\log\;(B_{\mathrm{ratio}\;\;}+0.001)\;∼\;\mathrm{Condition}+(1|\;\mathrm{Sample})\;,$$

The effect size and false discovery rate (FDR) for the condition coefficient were assessed. Significant coefficients (FDR < 0.05) indicated a change in cell proximity under HX conditions relative to NX, suggesting a disruption in cellular neighborhoods. A positive effect size indicated that cell type B is more densely clustered near cell type A (center cell) in HX compared with NX, suggesting increased proximity. In contrast, a negative effect size indicated that cell type B is closer to cell type A in NX compared with HX, indicating a relative decrease in proximity under hypoxic conditions.

### Differential gene expression analysis

To identify the differentially expressed genes (DEGs) between conditions and regions, we employed MAST (Finak et al., 2015) using Seurat's FindMarkers function on the raw counts matrix. Only genes expressed in a minimum of 5% of cells in either comparison group were tested. *p*-values were adjusted with the Benjamini–Hochberg correction method to obtain FDR values. Specific log_2_(FC) values are reported in Extended Data Table 5-1. Genes with a |log_2_(FC)| > 0.25 and FDR < 0.05 were considered significantly differentially expressed.

### Cell state scoring

To calculate cell state scores, we selected genes whose expression has been previously shown to be enriched in cells primed for OL differentiation (OL genesis score) or enriched in mature OLs [OL maturation score (Marques et al., 2016; Floriddia et al., 2020; Takeuchi et al., 2020)]; enriched in reactive astrocytes over homeostatic astrocytes [reactive astrocyte score (Zamanian et al., 2012; Liddelow et al., 2017; Matusova et al., 2023)]; and enriched in reactive microglia compared with homeostatic microglia (reactive microglia score; Paolicelli et al., 2022). Cell state scores were calculated for each cell from the normalized, integrated datasets by taking the average *z*-score for the genes in the cell state gene list in each cell. Then, cell state scores for specific cell groups (i.e., specific cell types or cell subtypes for each replicate for each anatomical region) were calculated by taking the average cell state score across all cells within that group of cells. To compare changes from NX to HX, statistical analysis was performed using the two-tailed, unpaired, Student's *t* test comparing the cell state scores from the three NX replicates to three HX replicates within each cell type and anatomical region analyzed. Genes used for the OL genesis score were *Ptprz1*, *Qk*, *Itpr2*, *Gpr17*, *Fyn*, and *Tcf7l2*. Genes used for the OL maturation score were *Mog*, *Mag*, *Cnp*, *Plp1*, *Myrf*, *Egr2*, *Fos*, *Fosb*, *Klk6*, *Ptgds*, *Car2*, *Grm3*, *Npsr1*, *Jph4*, *Aspa*, *Msmo1*, *Sqle*, *Hmgcs1*, *Idi1*, *Opalin*, and *Trf*. Genes used for the reactive astrocyte score were *Serpina3n*, *C3*, *Emp1*, *Gfap*, *Ggta1*, *H2-T23*, *Cd109*, *Hspb1*, and *lcn2*. Genes used for the reactive microglia score were *Lpl*, *Cst7*, *Ptprc*, *Trem2*, *Lgals3*, *Axl*, *Lyz2*, *Clec7a*, *Spp1*, *Cst7*, and *Apoe*.

### Ligand–receptor analysis

We performed ligand–receptor (LR) interaction analysis on the raw gene expression datasets using CellChat v2 (RRID:SCR_021946; Jin et al., 2023). For each experimental condition, spatial coordinates were aggregated, and an average spot size was computed to normalize the interaction distances. Coordinates from individual replicates were appropriately translated to prevent overlap and maintain the spatial integrity of the samples. The preprocessed data were then converted to a CellChat object, which encapsulated the gene expression matrix, cellular metadata, and spatial coordinates. In our analysis, we grouped cells based on both cell type and cell subtype classes and incorporated spatial data to contextualize cell interactions within the tissue architecture. We then aligned our gene expression data with established molecular communication networks to predict potential LR and in turn cell–cell interactions. The likelihood and extent of intercellular signaling interactions were estimated by assessing the expression of LR pairs. The truncated mean calculation for average gene expression per cell group and bootstrapping for statistical inference were performed to ensure estimation robustness. The communication probabilities were calculated, accounting for the effect of spatial distance on signaling potential by adjusting for the scaled interaction range and considering contact-based nearest neighbor interactions. Subsequently, pathways were aggregated, and centrality analysis was performed to identify key nodes within the communication network. To explore the differential intercellular communication networks between HX and NX, data from both conditions were integrated. Differential expression analysis was then carried out to identify genes that displayed significant changes in expression levels, with a threshold of *p* < 0.01. Changes in ligand and receptor expression level with a |log_2_(FC)| > 0.25 were considered significant.

### Code accessibility

The custom code for cell proximity analysis has been uploaded to GitHub: https://github.com/keon-arbabi/spatial_hypoxia/blob/main/keon_spatial_analyses.R.

10.1523/ENEURO.0224-24.2024.d1 Custom computational code Download Custom computational code, TXT file.

### Tissue preparation and immunofluorescence staining

Brains were harvested from P20 HX and NX exposed mice, fixed in 4% paraformaldehyde (PFA) for 24 h at 4°C, washed three times with PBS, stored in 30% sucrose in PBS for at least 48 h at 4°C, embedded in optimum cutting temperature (O.C.T.) mounting medium (Tissue-Tek), and stored at −80°C. Frozen embedded brains were sectioned coronally at 20 µm and stored at −80°C. For immunofluorescence staining, brain sections were first dried for 20 min at 37°C, washed three times with PBS, incubated in blocking solution (containing 10% BSA, 6% normal donkey serum, 0.1% Triton X-100 for 1 h at RT), and then incubated in primary antibody solution containing 1:500 anti-NCAN (Cedarlane Labs, catalog #AF5800-SP) diluted in blocking buffer overnight at 4°C. Sections were then washed three times with PBS-T (0.1% Triton X-100 in PBS) and incubated in Alexa Fluor 555–labeled secondary antibody solution (1:500 diluted in PBS-T, Thermo Fisher Scientific, catalog #A-21436) for 1 h at RT. Sections were then washed three times with 0.1% Triton X-100 and mounted with Fluoromount mounting medium with DAPI (Thermo Fisher Scientific, catalog #00-4959-52).

### Immunofluorescence imaging

Immunofluorescence-stained slides were scanned on a 3DHistech Pannoramic 250 Flash III Slide Scanner using a Zeiss 40 × 0.95 NA objective. The instrument was operated in extended focus mode (seven focal planes spanning a 5 μm axial distance) to capture the entire cell volume across each tissue section. The microscope is housed in the Imaging Facility at The Hospital for Sick Children in Toronto (ON, Canada).

### NCAN fluorescence intensity analysis

Immunofluorescence images were imported to the HALO Image Analysis Platform (Indica Labs, v.3.5.3577.173) for analysis. To quantify differences in NCAN distribution across the depth of the cortex, the cortex of each coronal section (from pia to corpus callosum) was manually segmented into three approximately equidistant regions encompassing the superficial cortical layers, middle cortical layers, and deep cortical layers. Average fluorescence intensity was quantified using the HALO Area Quantification FL module for each region.

## Results

**Figure 1.**
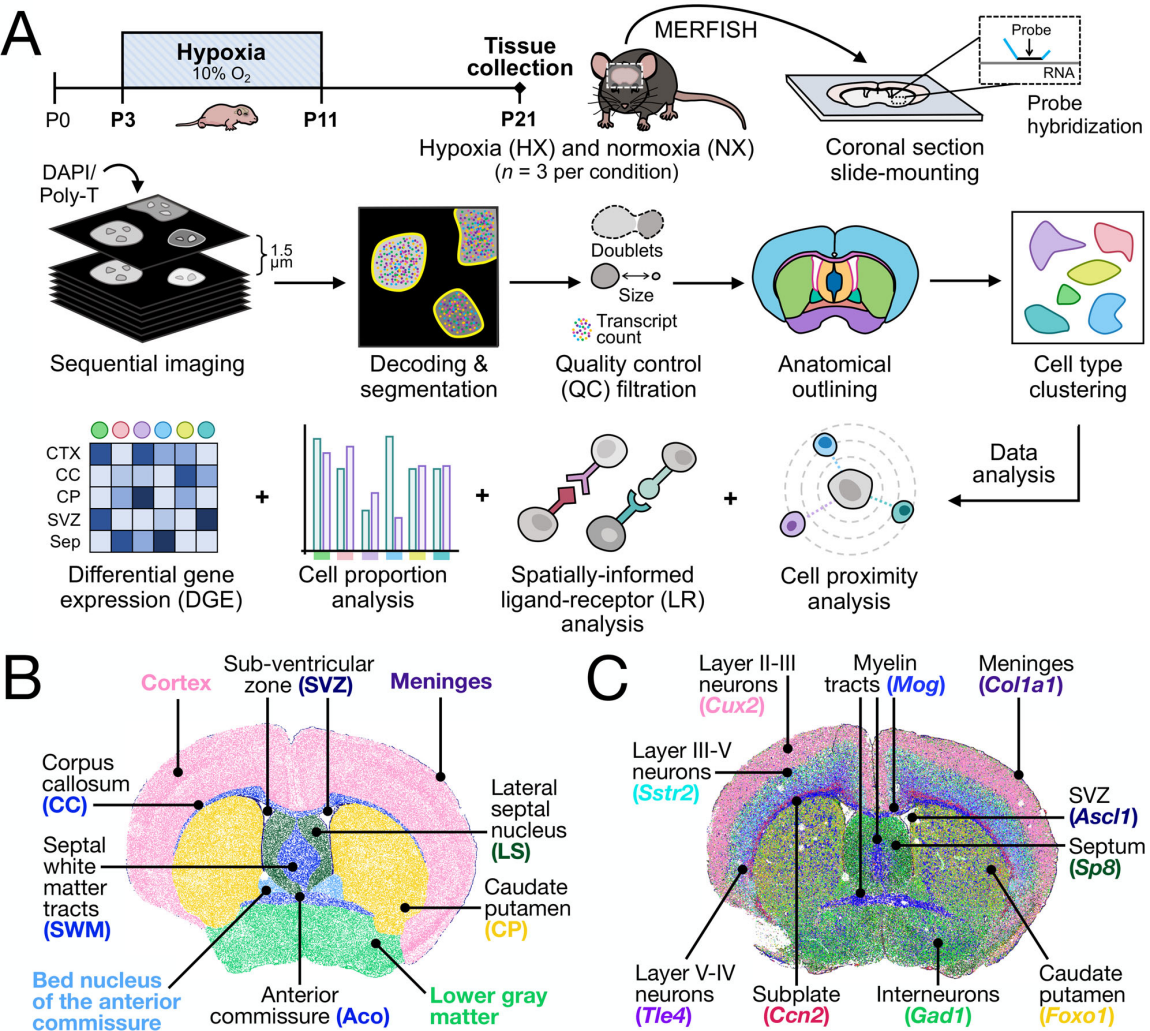
Multiplexed error-robust fluorescent in situ hybridization (MERFISH) sequencing identifies all major cell types and anatomical regions of the postnatal murine brain. ***A***, Schematic representation of the HX paradigm, tissue harvesting, tissue processing, quality control, and downstream data analysis approach applied to the MERFISH dataset. Tissue was harvested and processed for postnatal day 21 (P21) HX and NX mice (*n *= 3 male mice per condition). The MERFISH probe set used in this study targeted a curated panel of genes listed in Extended Data Table 1-1. MERFISH-derived spatial map displaying the (***B***) major anatomical regions outlined during sample processing steps and (***C***) a select set of genes used to identify and outline the anatomical regions utilized in downstream data analysis. Areas of the brain are colored according to their anatomical region or major identifying gene.

We employed a widely used mouse model of chronic sublethal HX to investigate the brain's regenerative response to neonatal injury (Fig. 1*A*, experimental schema). Mouse litters were exposed to 10% oxygen (HX) or 21% oxygen (normoxia, NX) between P3 and P11 (see Materials and Methods), a developmental period in mice that resembles the third gestational trimester in humans (Semple et al., 2013). Mouse dams were rotated daily between NX and HX conditions to avoid any confounding based on maternal care or nutrition. All mice were then moved to an environment with normal oxygen levels until they were euthanized at P21 (see Materials and Methods). We focused specifically on P21 as it represents a peak time during the postinjury repair period (Fagel et al., 2006; Salmaso et al., 2014; Forbes et al., 2020; Jablonska et al., 2022).

10.1523/ENEURO.0224-24.2024.t1-1 Table 1-1 **MERFISH gene panel**. Curated panel of 497 genes targeted for MERFISH. Download Table 1-1, XLS file.

We performed multiplexed error-robust fluorescence in situ hybridization (MERFISH) on coronal brain sections from three HX and three NX mice (see Materials and Methods). An RNA probe panel of 497 genes (Extended Data Table 1-1) was selected to enable the identification of major forebrain cell types as well as components of biologically relevant pathways including growth factors, wingless-related integration site (WNT)/β-catenin, bone morphogenic protein (BMP), immune recognition and regulation, perineuronal net components, and senescence-associated genes (see Materials and Methods). Cells were segmented using an established deep-learning algorithm, Cellpose 2.0 (Pachitariu and Stringer, 2022), to predict cell boundaries based on DAPI, a nuclear stain, and PolyT, an mRNA stain (see Materials and Methods). After segmentation, transcript counts were normalized by volume, and quality control filtering was performed to remove segmented cells with abnormal physical size, low total number of transcripts, and potential doublets (Fig. 1*A*, see Materials and Methods). The resulting dataset, encompassing all replicates and all regions, had a median volume of 179.5 µm^3^ per cell and a median expression of 285.6 transcripts per cell.

**Figure 2.**
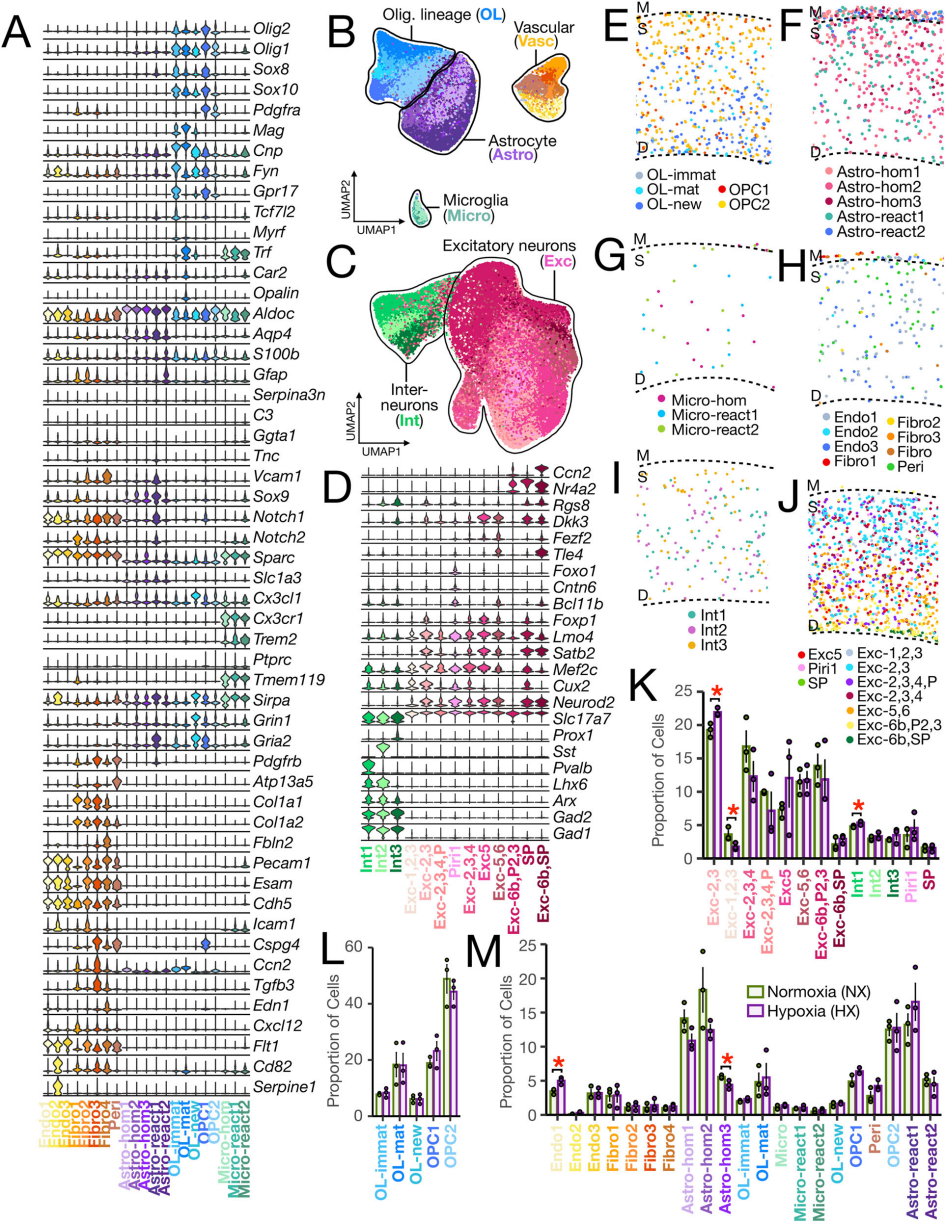
Cell type classification and spatial distribution in the P21 mouse cortex. Profiling of non-neuronal (***A***, ***B***) and neuronal (***C***, ***D***) cell types in the cortex for a total of 668,947 cells. Transcriptomic profiling for corpus callosum and subventricular zone (SVZ) cells is shown in Figure 3, and profiling of septum, caudate putamen (CP), anterior commissure (ACo), and septal white matter tracts (SWM) cells is shown in Extended Data Figure 2-1. Quality control metrics for each replicate are shown in Extended Data Figure 2-2. ***A***, Violin plots showing gene expression patterns of non-neuronal Cell Subtypes. UMAP visualization of (***B***) non-neuronal cells colored by Cell Subtype as in ***A*** and (***C***) neuronal cells colored by Cell Subtype as in ***D***. UMAP plots are generated from combined replicates across NX and HX conditions. Each dot represents a single cell, and cells are grouped with a solid line into major Cell Types. Each major Cell Type group was subsetted and clustered independently to allow identification of Cell Subtypes. UMAP visualizations of each Cell Type dataset, colored by Cell Subtype, are shown in Extended Data Figure 2-3. ***D***, Violin plot showing gene expression patterns of neuronal Cell Subtypes. ***A***, ***D***, Violin plots show several key canonical marker genes used for cluster identification. The width of the violin corresponds to the proportion of nuclei expressing the indicated gene, and the color of the violin corresponds to the Cell Subtype. Abbreviations for cell types are as follows: Exc, excitatory neuron; Int, interneuron; Exc-1,2,3, cortical layers I–III excitatory neurons; Exc-2,3, cortical layers II–III excitatory neurons; Exc-2,3,4, cortical layers II–IV excitatory neurons; Exc-2,3,4,P, cortical layers II–IV excitatory neurons and piriform neurons; Exc-5, cortical layer V excitatory neurons; Exc-5,6, cortical layers V–VI excitatory neurons; Exc-6b,P2,3, cortical layer VIb excitatory neurons and piriform neurons; Exc-6b,SP, cortical layer VI excitatory neurons and subplate neurons; Piri, piriform neurons; SP, subplate neurons; Vasc, vascular cell; OL, oligodendrocyte-lineage cell; Astro, astrocyte; Micro, microglia; OL-immat, immature oligodendrocyte; OL-mat, mature oligodendrocyte; OL-new, newly formed oligodendrocyte; OPC, oligodendrocyte precursor cell; Astro-hom, homeostatic astrocyte; Astro-react, reactive astrocyte; Micro-hom, homeostatic microglia; Micro-react, reactive microglia; Endo, endothelial cell; Fibro, fibroblast; Peri, pericyte. Spatial plots displaying the distribution of (***E***) OL-lineage cells, (***F***) astrocytes, (***G***) microglia, (***H***) vascular cells, (***I***) interneurons, and (***J***) excitatory neurons. Spatial plots are colored by Cell Subtype, dotted lines demarcate the cortex, and labels include deep part of the cortex, “d”; superficial part of the cortex, “s”; and meninges, “m.” Bar plots displaying the proportion of (***K***) neuronal, (***L***) OL-lineage, and (***M***), non-neuronal Cell Subtypes in the cortex of HX versus NX mice. The *x*-axis displays all major Cell Types and Cell Subtypes profiled through MERFISH. Data obtained from *n* = 3 male biological replicates per condition. Bars represent the average values for each condition, and dots represent the average values for each mouse per condition. Significance is determined using the two-tailed Student's *t* test. *p < 0.05; **p < 0.01. Comparisons not labeled with asterisks are not significant. Error bars represent the average ± 1 standard deviation. Each bar is labeled by Cell Subtype as previously defined.

**Figure 3.**
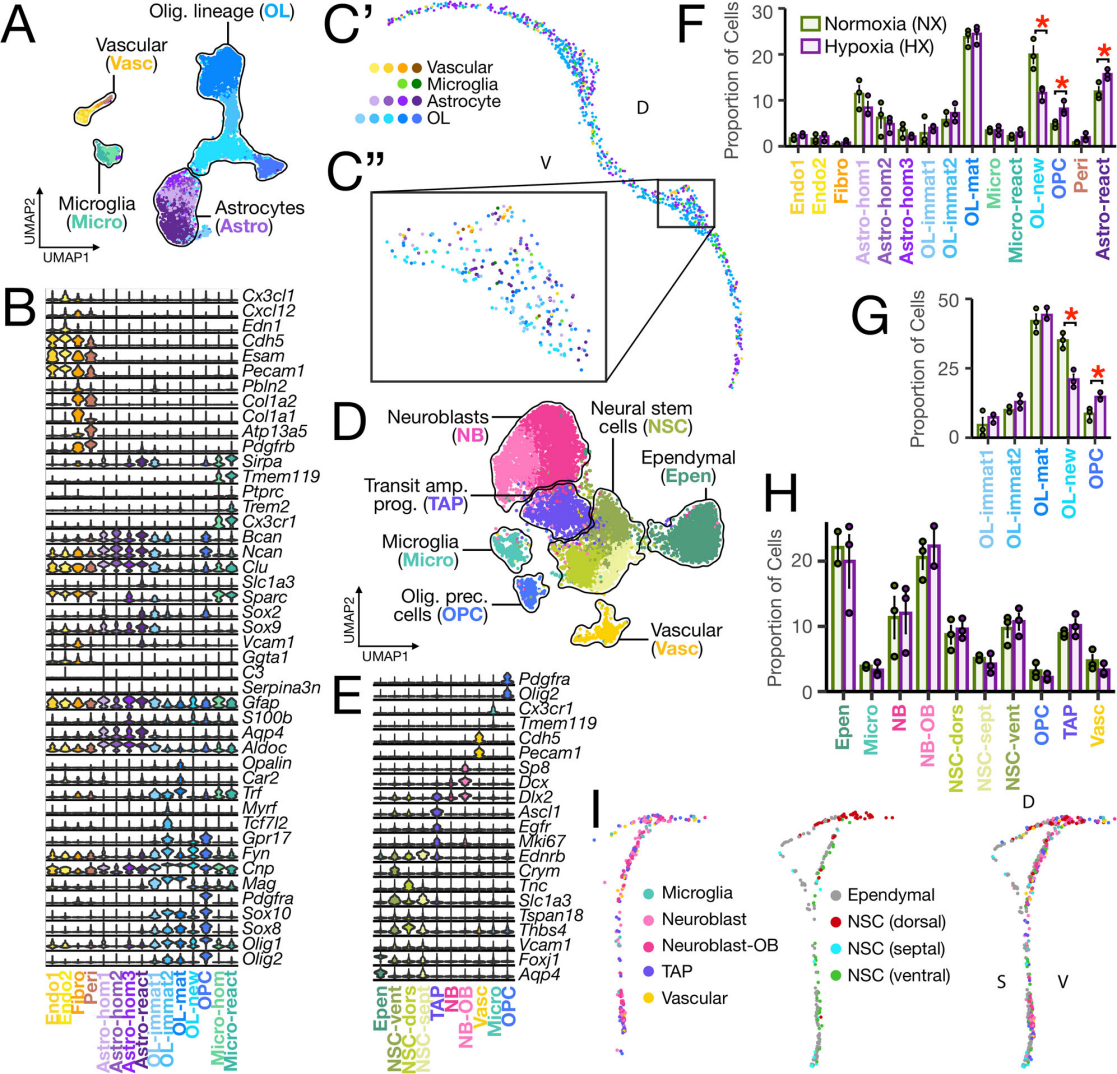
Cell type classification and spatial distribution in the P21 mouse SVZ and corpus callosum reveal arrested OL differentiation in the corpus callosum following neonatal hypoxia. ***A***, UMAP visualization of all corpus callosum cells, totaling 8,708 cells. UMAP plots are generated from combined replicates across NX and HX conditions. Quality control metrics for each replicate are shown in Extended Data Figure 2-2. Each dot represents a single cell, and cells are grouped with a solid line into major Cell Types: Astro, astrocyte; Micro, microglia; OL, oligodendrocyte; Vasc, vascular. Each major Cell Type group was subsetted and clustered independently to allow identification of Cell Subtypes. UMAP visualizations of each Cell Type dataset, colored by Cell SubType, are shown in Extended Data Figure 3-1. ***B***, Violin plot showing gene expression patterns of Cell Subtypes identified in the corpus callosum with several key canonical marker genes used for cluster identification. The width of the violin corresponds to the proportion of cells expressing the indicated gene, and the color of the violin corresponds to the cell subtype. Each column is labeled by Cell Subtype as previously defined. ***C*’**, Spatial plot displaying the distribution of Cell Types in the CC. ***C*”**, Zoomed view of the panel delineated in ***C*’**. ***D***, UMAP visualization of all SVZ cells, totaling 10,754 cells. UMAP plot is generated from combined replicates across NX and HX conditions. Quality control metrics for each replicate are shown in Extended Data Figure 2-2. Each cluster is colored by cell type and is labeled by cell type, with all NSCs labeled together and all neuroblasts labeled together, as follows: Epen, ependymal cell; TAP, transit-amplifying progenitor; NB, neuroblast; NB-OB, olfactory bulb neuroblast; NSC, neural stem cell; NSC (dors), dorsal neural stem cell; NSC (sept), septal neural stem cell; NSC (vent), ventral neural stem cell. ***E***, Violin plot showing gene expression patterns of Cell Subtypes identified in the SVZ with several key canonical marker genes used for cluster identification. The width of the violin corresponds to the proportion of cells expressing the indicated gene, and the color of the violin corresponds to the cell subtype. Each column is labeled by Cell Subtype as previously defined. Bar plots displaying the proportion of (***F***) Cell Subtypes and (***G***) OL-lineage Cell Subtypes of HX versus NX mice. ***H***, Bar plot displaying the proportion of Cell Subtypes in the SVZ of HX versus NX mice. The *x*-axis displays all major Cell Subtypes profiled through MERFISH. Data obtained from *n* = 3 male biological replicates per condition. Bars represent the average values for each condition, and dots represent the average values for each mouse per condition. Error bars represent the average ± 1 standard deviation. Each bar is labeled by cell subtype as previously defined. ***I***, Spatial plot displaying the distribution of Cell Subtypes in the SVZ. For ***C*** and ***I***, the label “d” denotes dorsal, “v” denotes ventral, and “s” denotes septal.

Each coronal MERFISH image of the mouse brain was labeled based on region-specific marker genes and anatomic landmarks to define regions for downstream analysis (Fig. 1*B,C*). After normalizing each sample, we independently integrated and analyzed cells from select regions including the cortex, corpus callosum, SVZ, caudate putamen (CP), lateral septal nucleus (LS), anterior commissure (Aco), and septal white matter tracts (SWM), across all datasets. Highly variable genes were used to compute principal components for uniform manifold approximation and projection (UMAP) and shared nearest neighbor clustering. After filtering, the final datasets included 668,947 cortex, 8,708 corpus callosum, 10,754 SVZ, 83,235 CP, 19,088 LS, 4,311 Aco, and 12,837 SWM cells across all samples, for a total of 807,880 transcriptionally profiled cells in these regions (Figs. 2*B,C*, 3*A,D*; Extended Data Fig. 2-1*A–D*). Our analyses focused on the cortex, corpus callosum, and SVZ, given their relevance to HX injury and ample cell numbers, contributing statistical power to our findings. The number of unique genes and transcripts per cell for these three regions are displayed in Extended Data Figure 2-2*A–F*.

10.1523/ENEURO.0224-24.2024.f2-1 Figure 2-1 **Cell Type identification across the P21 brain.** UMAP visualization of cell types identified in the **(A)** septum, **(B)** caudate putamen (CP), **(C)** anterior commissure (ACo), and **(D)** septal white matter tracts (SWM), where each dot represents a single cell. UMAP plots are generated from combined replicates across NX and HX conditions. Each cluster is colored by Cell Type. Download Figure 2-1, TIF file.

10.1523/ENEURO.0224-24.2024.f2-2 Figure 2-2 **Quality control metrics of the MERFISH data.** Violin plots showing select quality control parameters used to profile the data, including the number of unique genes per cell in the **(D)** cortex, **(E)** corpus callosum, and **(F)** SVZ, and the number of transcripts per cell in the **(G)** cortex, **(H)** corpus callosum, and **(I)** SVZ. Download Figure 2-2, TIF file.

10.1523/ENEURO.0224-24.2024.f2-3 Figure 2-3 **Cell Subtype identification in the cortex of the P21 brain. (A-D, F-G)** UMAP visualization of cortical **(A)** interneurons and **(B)** excitatory neurons, **(C)** OL-lineage cells, **(D)** astrocytes, **(F)** microglia, and **(G)** vascular cells. UMAP plots are generated from combined replicates across NX and HX conditions. Each dot represents a single cell, and clusters of cells are colored by Cell Subtype as previously defined. **(E)** Dotplot displaying average expression level of reactive astrocyte genes in all astrocyte Cell Subtypes in (D). The dotplot is intended to clarify the data shown in Figure 2A where the range of gene expression in the violin plots are too wide to visually depict the average expression level of these genes. Dot size indicates the percentage of cells in the group where the gene is detected, and color indicates average expression level of the gene within the group. Download Figure 2-3, TIF file.

10.1523/ENEURO.0224-24.2024.f3-1 Figure 3-1 **Cell Subtype identification across the cortex, SVZ, and corpus callosum of the P21 brain. (A-B, D-F)** UMAP visualization of corpus callosum **(A)** OL-lineage cells, **(B)** astrocytes, **(D)** microglia, **(E)** vascular cells; and SVZ **(F)** neural stem cells (NSC). UMAP plots are generated from combined replicates across NX and HX conditions. Each dot represents a single cell, and clusters of cells are colored by Cell Subtype as previously defined. **(C)** Dotplot displaying average expression level of reactive astrocyte genes in all astrocyte Cell Subtypes in (B). This dotplot is intended to clarify the data shown in Figure 3B where the range of gene expression in the violin plots are too wide to visually depict the average expression level of these genes. Dot size indicates the percentage of cells in the group where the gene is detected, and color indicates average expression level of the gene within the group. Download Figure 3-1, TIF file.

### Cell type identification by MERFISH

A major benefit of high-resolution spatial transcriptomics is the potential to characterize cell type diversity within defined anatomical borders. As such, we analyzed each major anatomic region independently to ascertain region-specific cell type composition and spatially restricted cell types. In the cortex and corpus callosum, we classified cells at two levels of cell type resolution. First, we identified broad cell type categories (referred to as “Cell Types”), and then each Cell Type group was further subclustered to identify more specific subtypes or region-specific cell states (the “Cell Subtypes”). SVZ cells were classified at one level of resolution which included mostly broad cell type categorizations with the exception of neural stem cell (NSC) subtypes. Other regions such as the CP, LS, ACo, and SWM, which were not analyzed in depth in this paper, were classified at one level of resolution with broad categories using the same criteria described below for Cell Types in the cortex and corpus callosum. Unless otherwise specified, genes used for characterizing cell types were selected from well-known and widely used brain cell type markers.

For cortex and corpus callosum, Cell Types included non-neuronal cells corresponding to OL-lineage cells, vascular and perivascular cells (from here on collectively termed vascular cells), astrocytes, microglia, and neuronal cells present only in the cortex, corresponding to excitatory neurons and interneurons. These included clusters of OL-lineage cells identified with markers such as *Olig1*, *Olig2*, *Sox8*, and *Sox10*; clusters of astrocytes identified with *Aqp4* and *S100b*; clusters of vascular cells identified with at least one of *Pecam1*, *Col1a1*, or *Pdgfrb*; and microglia identified with *Cx3cr1* (Fig. 2*A,B* for cortex and Fig. 3*A,B* for corpus callosum). In the cortex only, clusters of excitatory neurons were identified with high expression of *Slc17a7* along with some other excitatory neuron genes like *Neurod2* and the layer-specific markers described later while interneurons were identified with *Gad1* and *Gad2* (Fig. 2*C,D*).

Next, each Cell Type group in the cortex and corpus callosum was subsetted and independently clustered to identify distinct clusters for subclassification into Cell Subtypes. In the cortex, interneurons formed three subtype clusters (Extended Data Fig. 2-3*A*), each enriched for known markers of interneuron subpopulations: *Pvalb* (Int 1), *Sst* (Int 2), and *Prox1* (Int 3; Fig. 2*D*). Int1 and Int2 were spatially concentrated in middle to deeper cortical layers whereas Int3 was concentrated in upper cortical layers (Fig. 2*I*). Excitatory neurons formed 10 clusters (Extended Data Fig. 2-3*B*) which were annotated based on their spatial distribution in the cortex (Fig. 2*J*) and expression of known cortical layer markers (Fig. 2*D*). These included a cluster of cells that were spatially concentrated in cortical layers I–III and expressed *Cux2* and *Mef2c* (Exc-1,2,3); a cluster of cells spatially concentrated in cortical layers II and III which expressed high *Cux2*, *Mef2c*, *Satb2*, and *Lmo4* (Exc-2,3); a cluster of cells spatially concentrated in cortical layers II–IV and expressed *Mef2c* and *Lmo4* (Exc-2,3,4); a cluster of cells spatially concentrated in cortical layers II–IV and all three layers of the piriform cortex and expressed low levels of *Mef2c* and *Lmo4* (Exc-2,3,4,P); a cluster of cells spatially concentrated in piriform layer 1 and expressing *Mef2c*, *Lmo4*, *Bcl11b*, *Foxo1*, and *Cntn6* (Piri1); a cluster of cells spatially concentrated in cortical layers V and expressing *Dkk3*, *Foxp1*, *Lmo4*, *Satb2*, and *Mef2c* (Exc5); a cluster of cells spatially concentrated in cortical layers V and VI and expressing *Tle4*, *Dkk3*, and *Fezf2* (Exc-5,6); a cluster of cells spatially concentrated in cortical layer VIb and piriform layers 2 and 3 and expressing *Nr4a2* (Exc-6b,P2,3); a cluster of cells spatially concentrated in cortical layers 6b and the subplate and expressing *Ccn2*, *Tle4*, *Nr4a2*, *Satb2*, and *Rgs8* (Exc-6b,SP); and a cluster of cells spatially concentrated in the subplate and expressing *Nr4a2 Dkk3*, *Lmo4*, *Satb2*, *Mef2c*, and *Cux2* (SP; Extended Data Fig. 2-3*B* and Fig. 2*D,J*).

OL-lineage cell clusters in the cortex (Fig. 2*A*) and corpus callosum (Fig. 3*B*) were classified as OPCs if they expressed *Pdgfra* and OLs if they expressed *Mag* and no *Pdgfra*. OPCs formed one distinct cluster in the corpus callosum (Extended Data Fig. 3-1*A*), but two clusters in the cortex (Extended Data Fig. 2-3*C*). In both regions, OLs formed multiple clusters corresponding to different stages of lineage progression with graded expression patterns of the canonical OL marker *Mag* and other genes described in previous studies as necessary for and upregulated at different stages of OL development. OL clusters with the lowest expression of Mag and moderate to high enrichment of OL differentiation and early marker genes *Fyn* (Osterhout et al., 1999) and *Gpr17* (Fumagalli et al., 2011) were classified as newly formed OLs (OL-new); clusters with high enrichment for *Fyn*, *Gpr17*, and other OL differentiation genes such as *Tcf7l2* (Guo and Wang, 2023) and *Myrf* (Bujalka et al., 2013) were classified as immature OLs (OL-immat); and clusters with the highest expression of *Mag* and enriched for mature, myelinating OL genes *Trf* (Bujalka et al., 2013), *Car2* (Floriddia et al., 2020), and *Opalin* (Marques et al., 2016), were classified as mature OLs (Fig. 2*A* and Extended Data Fig. 2-3*C* for cortex and Fig. 3*B* and Extended Data Fig. 3-1*A* for corpus callosum). OLs in the cortex, particularly OL-immat and OL-mat, were enriched in the deeper layers (Fig. 2*E*). In the corpus callosum, OL-new was enriched ventrally (Fig. 3*C*’*,C*”), in regions adjacent to the SVZ. This suggests that these newly formed OLs appear to migrate from the SVZ toward the corpus callosum.

Astrocyte clusters were subclassified as reactive astrocytes if they expressed high *Gfap* and were differentially enriched for at least two of the reactive astrocyte markers *Serpina3n*, *Ggta1* (Liddelow et al., 2017), and *C3* (Clarke et al., 2018) and classified as homeostatic astrocytes otherwise in both the cortex (Fig. 2*A* and, for better visualization, Extended Data Fig. 2-3*E*) and corpus callosum (Fig. 3*B* and Extended Data Fig. 3-1*C*). In the cortex, reactive astrocytes formed two clusters (Extended Data Fig. 2-3*D*), with one population (Astro-react1) distributed throughout the cortex and meninges, and the second population (Astro-react2) concentrated in the meninges (Fig. 2*F*). Analysis of differentially expressed genes (DEGs) between these two clusters revealed that Astro-react1 was enriched for *Gria2 Vcam1*, *Sox9*, and *Notch1/2*, and Astro-react2 was enriched for *Gfap*, *S100b*, and *Sparc* (Fig. 2*A*). In the corpus callosum, we identified one cluster of reactive astrocytes (Astro-react) which, like Astro-react1 of the cortex, differed from the meninge-enriched astrocytes with their high expression of *Vcam1* and *Sox9* (Fig. 3*B* and Extended Data Fig. 3-1*B*). In both the cortex (Fig. 2*A* and Extended Data Fig. 2-3*D*) and corpus callosum (Fig. 3*B* and Extended Data Fig. 3-1*B*), we identified three clusters of homeostatic astrocytes (Astro-hom1, Astro-hom2, Astro-hom3). Similar naming of these clusters in the two regions does not imply transcriptomic similarity.

Microglia clusters in the cortex (Fig. 2*A*) and corpus callosum (Fig. 3*B*) were subclassified as reactive microglia if they expressed high levels of the reactive microglia marker *Trem2* or subclassified as homeostatic microglia otherwise. Cortical microglia formed one homeostatic cluster (Micro-hom) and two reactive clusters (Extended Data Fig. 2-3*F*) whereas corpus callosum microglia formed one homeostatic (Micro-hom) and one reactive cluster (Micro-react; Extended Data Fig. 3-1*D*). Microglia subtypes were distributed throughout the cortical layers (Fig. 2*G*) and corpus callosum (Fig. 3*C*’*,C*”).

Vascular cell clusters in the cortex (Fig. 2*A*) and corpus callosum (Fig. 3*B*) were subclassified as pericytes (Peri) if they expressed high levels of pericyte markers *Pdgfrb* and *Atp13a5*, fibroblasts (Fibro) if they expressed high levels of fibroblast markers *Col1a1* and *Col1a2*, and endothelial cells if they expressed high levels of endothelial cell markers *Pecam1*, *Esam*, and *Cdh5* and low levels of pericyte and fibroblast markers. Pericytes formed only one cluster in both the cortex (Extended Data Fig. 2-3*G*) and corpus callosum (Extended Data Fig. 3-1*E*). In the cortex, fibroblasts formed four clusters (Extended Data Fig. 2-3*G*). In general, there was a higher density of fibroblasts in the meninges compared with the cortex, but one cluster in particular. Fibro-1 was highly concentrated in the meninges (Fig. 2*H*). Compared to the other fibroblast clusters, Fibro-1 was differentially enriched for genes like *Cxcl12*, *Gfap*, and *Pdgfra* (Fig. 2*A*). In the corpus callosum, fibroblasts formed one distinct cluster (Extended Data Fig. 3-1*E*). Endothelial cells formed three clusters in the cortex (Extended Data Fig. 2-3*G*) and formed two clusters in the corpus callosum (Extended Data Fig. 3-1*E*).

In the SVZ, we identified vascular cells, microglia, ependymal cells, slowly proliferating neural stem cells (NSCs), transit-amplifying progenitors (TAPs), OPCs, and neuroblasts (Fig. 3*D*). Slowly proliferating NSCs were identified by their expression of known and recently established SVZ NSC markers *Vcam1*, *Thbs4*, *Tspan18*, and *Slc1a3* (Borrett et al., 2020) and absence of *Mki67*; TAPs by their expression of *Mki67*, *Egfr*, *Ascl1*, and *Dlx2*; neuroblasts by their expression of *Dlx2* and *Dcx* and absence of *Ascl1*; and ependymal cells by their expression of *Foxj1* (Fig. 3*E*). Iterative clustering of NSCs revealed three clusters (Extended Data Fig. 3-1*F*): one cluster was enriched for *Crym*, a ventral SVZ (ganglionic eminence) marker (Borrett et al., 2020), and spatially enriched along the ventrolateral wall of the SVZ (NSC-vent); one cluster was enriched for the known NSC gene *Tnc* and spatially enriched along the dorsal wall of the ventricle (NSC-dors); and the third cluster was enriched for *Ednrb*, a recently characterized SVZ NSC gene (Yuzwa et al., 2017), and spatially enriched along the septal wall of the ventricle (NSC-sept; Fig. 3*E,I*). Of the two neuroblast clusters, one was classified as olfactory bulb neuroblasts (NB-OB) for its expression of *Sp8*, a marker that is prominent in most neuroblasts destined for the olfactory bulb (Gaborieau et al., 2018; Fig. 3*E*). The other neuroblast cluster (NB) may represent those that will migrate either to the olfactory bulb or to the caudoputamen (De Marchis et al., 2004; Bordiuk et al., 2014).

### Region-specific patterns of signaling-related gene expression in the NX brain

Recent studies have suggested that cellular phenotypes are at least partly informed by spatial location in the brain (Zhang et al., 2021; Stogsdill et al., 2022; Allen et al., 2023). Before investigating how HX alters region-specific cellular signaling profiles, we first compared transcriptional profiles of non-neuronal cells between distinct brain regions (SVZ, corpus callosum, superficial layers of the cortex, and deep layers of the cortex) in the P21 NX brain to identify baseline regional heterogeneity. This was done by differential gene expression analysis where a false discovery rate (FDR) < 0.05 and log_2_ fold-change [log_2_(FC)] > 0.25 were used to identify statistically significant differences in each comparison (see Materials and Methods). We observed extensive differences in signaling-related gene expression levels across all regions and cell types.

Notably, vascular cells were remarkably different between the upper and deep cortical layers with respect to the expression of genes involved in WNT, insulin-like growth factor (IGF), BMP, and fibroblast growth factor (FGF) signaling. Upper cortex vascular cells were enriched for expression of *Fzd1/2/7*, *Wnt4/5a*, *Sfrp1*, *Igf2*, *Igfbp2*, *Bmp4/5/7*, *Fgf1*, *Fgfr1*, and *Fgfr2*, while deep cortex vascular cells were enriched for expression of *Igf1r* and *Apc* (Extended Data Fig. 4*F* and Extended Data Table 5-1). Compared to the corpus callosum and SVZ, vascular cells of the upper cortex but not the deep cortex were enriched for WNT ligand (*Wnt5a*), BMP ligand (*Bmp4/5/7*), and IGF ligand (*Igf2*, *Igfbp2*) genes (Extended Data Fig. 4*B–F* and Extended Data Table 5-1). These results suggest activation of the canonical WNT pathway within vascular cells in the upper cortex, and robust WNT inhibition mediated by the negative regulator *Apc* within vascular cells in the deep cortex of the NX brain.

OPCs in different regions exhibited unique patterns of signaling-related gene expression. For example, SVZ and corpus callosum OPCs expressed higher levels of *Bmp4* and *Notch1*, while cortical OPCs expressed higher levels of *Cx3cl1* (Extended Data Table 5-1). Microglia also showed notable differences between regions in the NX brain. SVZ microglia exhibited higher expression of genes involved in WNT signaling, such as *Fzd1/2/5/8*, *Lrp4/6/8*, and *Sfrp1*; genes involved in sonic hedgehog (SHH) signaling such as *Ptch1* and *Gli1/3*; and ephrin signaling genes such as *Efna2*, *Efnb2/3*, *Epha4*, and *Ephb1* compared with the corpus callosum, deep cortex, and upper cortex (Extended Data Fig. 4*A–C* and Extended Data Table 5-1). This may suggest that in the postnatal mouse brain, microglia in the SVZ are uniquely suited for sensing and regulating the SVZ niche to modulate neurogenesis (Ribeiro Xavier et al., 2015).

### HX-associated changes in cellular composition

We next assessed differences in global cell type proportions between HX and NX by leveraging the multiple biological replicates for each condition (see Materials and Methods). In the corpus callosum, we found that the proportion of newly formed OLs was markedly reduced following HX (42% reduction, *p* = 0.016), while the proportion of OPCs was significantly increased (72% increase, *p* = 0.025; Fig. 3*F,G*). These findings corroborate the idea that chronic sublethal neonatal HX induces WMI followed by a robust period of oligodendrogenesis (Fagel et al., 2006; Jablonska et al., 2022). However, we did not detect a significant change in the proportion of any OL-lineage Cell Subtypes in the cortex (Fig. 2*L*). This could imply that OL-lineage cells in the cortex are less severely affected by HX.

We also observed a significant increase in the proportion of reactive astrocytes in the corpus callosum in HX (32% increase, *p* = 0.037, Fig. 3*F*). This is consistent with previous work showing that there is an increase in the number of *C3*-expressing reactive astrocytes in the corpus callosum following acute perinatal WMI (Back and Rosenberg, 2014; Renz et al., 2022). In the cortex, we did not observe a significant change in the proportion of reactive astrocytes, but the proportion of Astro-hom3 was significantly reduced (21% reduction, *p* = 0.044).

In the cortex of HX mice, we identified a significant increase in the abundance of Endo-1 cells, the most abundant subpopulation of endothelial cells (45% increase, *p* = 0.015, Fig. 2*M*), suggestive of vascular remodeling.

Lastly, we observed a significant increase in the proportion of a large population of excitatory neurons in layers II–III (14% increase, *p* = 0.019) and a significant decrease in a small population of excitatory neurons in layers I–III (51% reduction, *p* = 0.049, Fig. 2*K*), indicating a change in the cell type composition of upper layer excitatory neurons. We also observed an increase in the proportion of Int1, a population of cortical interneurons (12% increase, *p* = 0.038, Fig. 2*K*). An increase in cortical neurons could suggest a disruption or delay in programmed neuron death and clearing in the middle-upper cortical layers (the last layers to form and mature), a process that largely takes place in the first week after birth, coinciding with the timing of HX exposure (Pfisterer and Khodosevich, 2017; Castillo-Ruiz et al., 2020). No significant change was observed in the proportion of SVZ cell types (Fig. 3*H*).

### Proximity analysis reveals widespread HX-associated changes in local cellular architecture

We next evaluated changes in local cellular neighborhood composition associated with neonatal HX. Unlike spot-based spatial transcriptomics methods, MERFISH enables us to accurately map both spatial locations and transcriptional identities of individual brain cells. We leveraged this information using a novel computational approach for quantifying condition-associated differences in local cell type proximities (see Materials and Methods), which addresses some limitations of existing methods (Dries et al., 2021; Palla et al., 2022). Briefly, we calculated the condition-related differences in the proportion of cells of one type in the vicinity of a second type, thus capturing asymmetrical differences in local proportions between cell types. For example, our approach can detect the depletion of astrocytes surrounding neurons when the reverse is not true. Second, we directly compare cell type proximities between conditions using a general linear mixed-effects model, accounting for the statistical nonindependence of measures from the same tissue sample when there are multiple biological replicates (see Materials and Methods). We applied this approach at the microscale, capturing local changes in proximity that matter for cellular interaction, however, we note that such differences may also reflect global cellular abundance differences between conditions (Figs. 2*K–M*, 3*F–H*).

**Figure 4.**
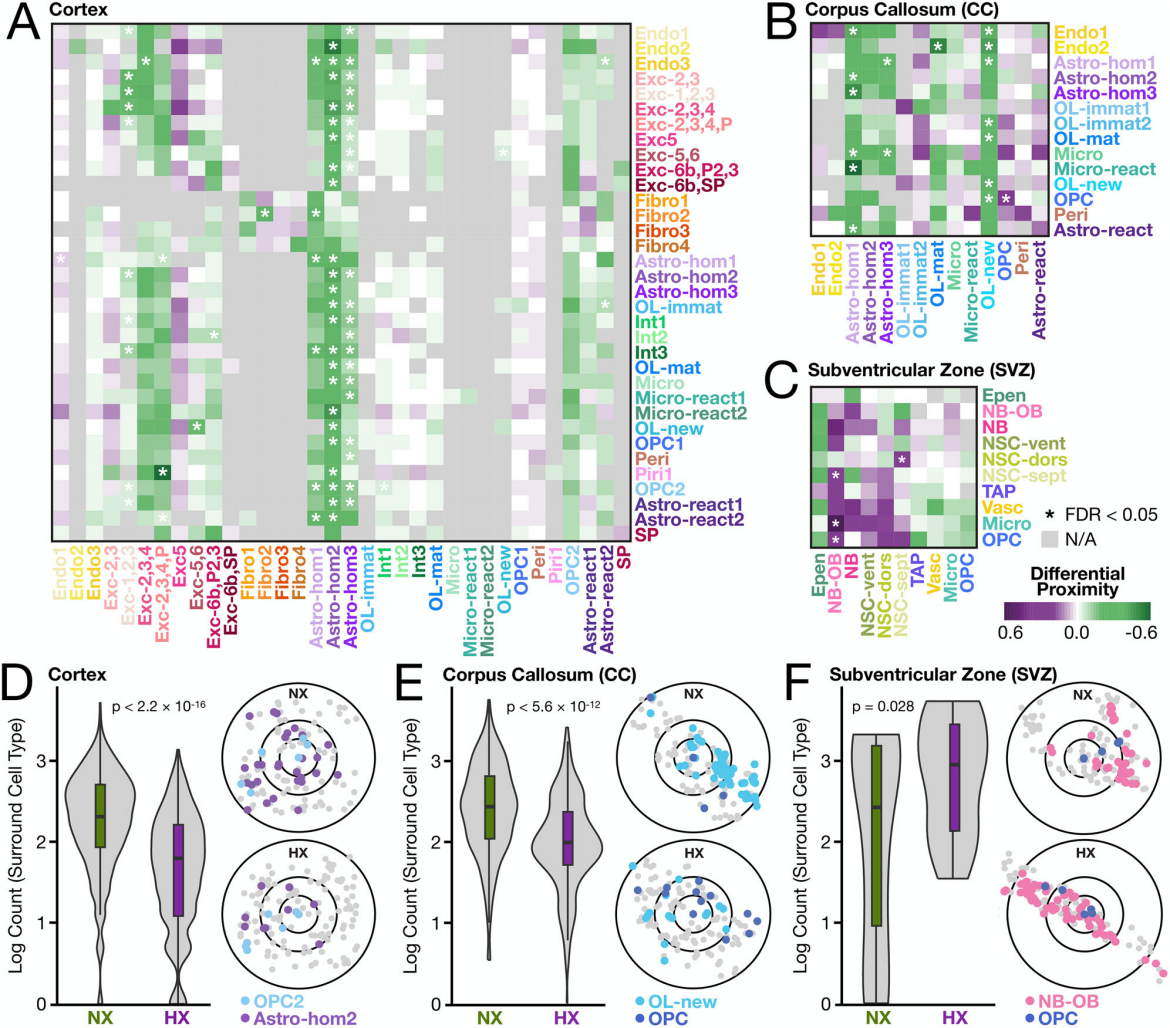
Neonatal hypoxia alters local spatial proximity between select Cell Type pairs across brain regions. Differential proximities between cell type pairs (heatmaps) in the (***A***) cortex, (***B***) corpus callosum, and (***C***) SVZ. For cortex and corpus callosum, Cell Subtype classification was used. Positive scores (purple) indicate an increased proportion of the surrounding cell type (columns, *x*-axis) in proximity of the center cell type (rows, *y*-axis) in HX relative to NX, whereas negative scores (green) indicate a decrease. Gray indicates insufficient nonzero data was present for the respective cell type pair. Significant (FDR < 0.05) relationships are denoted with an asterisk. Exemplar spatial plots (right panels) indicate the distribution of select center and surround cell types between NX (top) and HX (bottom) in the (***D***) cortex, (***E***) corpus callosum, and (***F***) SVZ. The outer circles in the spatial plots correspond to the radius used to calculate the differential proximities in ***A–C*** and have been normalized to reflect the median cellular distances per sample. Violin plots (left panels) show the counts of the surrounding cell type in proximity to every central cell across the sample corresponding to the spatial plot (not just the sample area shown). *p*-values for *t* tests are shown.

In the cortex, neonatal HX was associated with decreased proximity of homeostatic astrocytes to several cell types (Fig. 4*A*), including OPC2 (*β* = −0.46, FDR = 8.45 × 10^−5^; Fig. 4*D*), newly formed OLs (*β* = −0.48; FDR = 0.012), and immature OLs (*β* = −0.43, FDR = 4.21 × 10^−3^). This may indicate a disruption in the supportive interactions provided by astrocytes, such as the secretion of local factors (e.g., BDNF and CNTF) that promote OL maturation and myelination during healthy conditions and following injury (Miyamoto et al., 2015). HX induced dramatic changes in the proximity of different populations of cortical excitatory neurons to other neurons and non-neuronal cells. For example, after HX, a population of upper layer excitatory neurons, Exc-1,2,3, had decreased proximity to interneurons (Int-3, *β* = −0.15, FDR = 6.65 × 10^−6^), reactive (*β* = −0.06, FDR = 0.046) and homeostatic astrocytes (*β* = −0.13, FDR = 1.07 × 10^−3^), and OPC2 (*β* = −0.05, FDR = 1.93 × 10^−3^; Fig. 4*A*).

In the corpus callosum following HX (Fig. 4*B*), we observed significantly reduced proximity of newly formed OLs in the neighborhoods of several cell types, including OPCs (*β* = −0.37, FDR = 6.82 × 10^−3^; Fig. 4*E*) and endothelial cells (*β* = −0.65, FDR = 0.011). Additionally, we observed reduced proximity of surrounding reactive astrocytes (*β* = −0.57, FDR = 2.62 × 10^−13^) and microglia (*β* = −0.45, FDR = 0.016) near homeostatic astrocytes. Such local interactions are likely essential for OL maturation and efficient myelination (Miyamoto et al., 2015). The altered spatial organization of these cells potentially contributes to the impaired myelination characteristic of diffuse WMI.

In the post-HX SVZ, NSCs showed significantly increased proximity to differentiating neuroblasts (*β* = 0.28, FDR = 7.12 × 10^−3^), with a trending increase in proximity to OPCs, microglia, and vasculature (Fig. 4*C,F*). These changes align with the roles of activated microglia in promoting oligodendrogenesis and neurogenesis. The closer association with vascular components may indicate an adaptive response to HX, possibly involving angiogenesis or enhanced metabolic support. Neuroblasts saw increased proximity to NSCs (*β* = 0.36, FDR = 1.61 × 10^−4^), microglia (*β* = 0.81, FDR = 6.90 × 10^−4^), and OPCs (*β* = 0.54, FDR = 0.026). These changes could reflect neural repair through enhanced interactions of neuroblasts with NSCs for proliferation, with microglia for inflammation resolution, and with OPCs for myelination, altogether accelerating recovery processes. Overall, these observations suggest a reorganization or adaptation of the SVZ niche in response to neonatal HX, highlighting the region's plasticity under developmental stress.

In total, our proximity analysis emphasizes a crucial spatial component to the effects of neonatal HX beyond gene expression. Changes were extensive and heterogeneous across cell types and regions, ranging from disrupted myelination in the corpus callosum to adaptive cellular reorganization in the SVZ.

### Neonatal HX alters regional patterns of signaling-related gene expression

**Figure 5.**
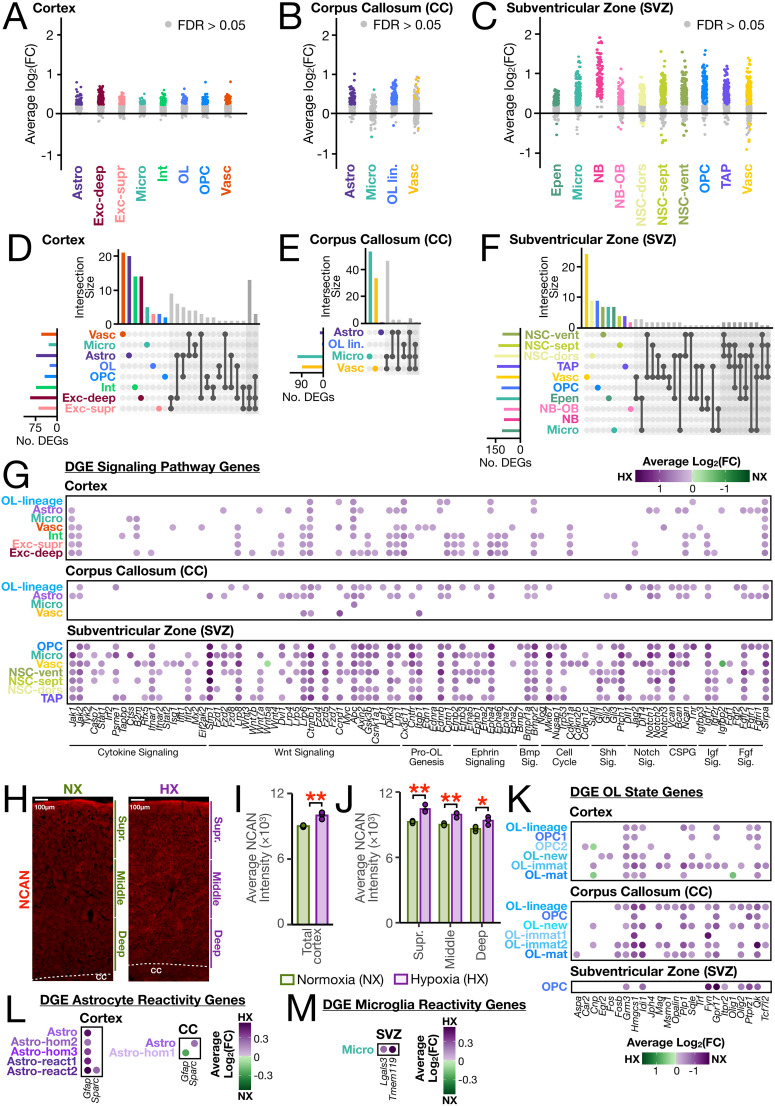
Differential gene expression analysis finds transcriptionally distinct cellular subpopulations in the HX brain. ***A–C***, Strip plots displaying DEGs between P21 HX and NX mice. Each dot represents a DEG: colored dots represent significant genes (FDR < 0.05) per cell type, and gray dots represent nonsignificant genes (FDR > 0.05). The *x*-axis displays all major cell types across the (***A***) cortex, (***B***) corpus callosum, and (***C***) SVZ regions profiled through MERFISH analysis. Data obtained from *n* = 3 biological replicates per condition. ***D–F***, UpSet plots displaying the number of unique and shared DEGs across cell types in the (***D***) cortex, (***E***) corpus callosum, and (***F***) SVZ. Unique genes are colored based on cell type, and genes shared between two or three cell types are indicated by black dots connected by lines according to shared origins. The horizontal histogram indicates the number of significant DEGs for each cell type, and the vertical bar plots show the number of significant DEGs (FDR < 0.05) specific to one, two, or three cell types. ***G***, Differential gene expression comparison of HX and NX in each region (cortex, corpus callosum, and SVZ) for signaling genes across Cell Types. ***H***, Representative images of NCAN immunofluorescence-stained P21 NX and HX cortices from coronal slices. The cortex of each slice was manually segmented into three approximately equidistant regions encompassing the superficial (supr.) cortical layers, middle cortical layers, and deep cortical layers. Dashed lines demarcate the corpus callosum, “CC.” ***I***, ***J***, Average positive fluorescence intensity of NCAN across the area of the (***I***) entire cortex and (***J***) each of the three segmented regions in ***H***. ***K***, ***L***, Differential gene expression comparison of HX and NX in each region (cortex, corpus callosum, and SVZ) for (***K***) OL-state genes across all OL-lineage cells (from Cell Type) and Cell Subtypes within the OL lineage, (***L***) astrocyte reactivity genes across all astrocytes (Astro from Cell Type) and Cell Subtypes within astrocytes, and (***M***) microglia reactivity genes across all microglia (Micro from Cell Type) and Cell Subtypes within microglia. Genes in ***K*** are grouped and labeled according to the signaling pathway or function that they participate in. Cortex and corpus callosum data in ***A***, ***B***, ***D***, ***E***, and ***G*** display Cell Type information and in ***K*** and ***L*** both Cell Type and Cell Subtype information. Only significant genes are shown. Colors represent average log_2_(FC) as per legend. Differential gene expression results comparing signaling genes from one anatomical region to another within the NX brain are shown in Extended Data Figure 5-1. Full differential gene expression results, including genes not shown here, are shown in Extended Data Table 5-1.

We next analyzed cell type- and region-specific transcriptional changes between HX and NX conditions using differential gene expression analysis (see Materials and Methods). This comparative analysis aimed to identify molecular processes perturbed during HX exposure in the context of early brain development. In all cell type populations assayed in the SVZ, corpus callosum, and cortex, we found that the vast majority of significant DEGs were upregulated following HX (Fig. 5*A–C* and Extended Data Table 5-1). While many genes exhibited widespread cell type–specific changes, many cells also shared DGEs within the forebrain regions (Fig. 5*D–F*).

10.1523/ENEURO.0224-24.2024.f5-1 Figure 5-1 **Comparison of signaling-related gene expression between anatomical regions in mice exposed to normoxic conditions.** Dotplots displaying differential gene expression results comparing Cell Types between SVZ, corpus callosum, deep cortex, and upper cortex within NX mice. Only significant genes (FDR < 0.05) are shown. The color of each dot represents the average log_2_(FC) as per each associated legend. Comparisons shown are **(A)** SVZ versus corpus callosum, **(B)** SVZ and deep cortex, **(C)** SVZ versus upper cortex, **(D)** corpus callosum versus deep cortex, **(E)** corpus callosum versus upper cortex, and **(F)** deep cortex versus upper cortex. Full results, including genes not depicted in these graphs, are shown in Table 5-1. Download Figure 5-1, TIF file.

10.1523/ENEURO.0224-24.2024.t5-1 Table 5-1 **Differential gene expression analysis within and between regions**. This table is available at available at https://figshare.com/s/f096946cf6543f034528. Differential gene expression analysis for several comparisons: HX versus NX differences within each Cell Type in each region, HX versus NX differences within each Cell Subtype in each region, region versus region comparisons within each Cell Type and within either HX or NX, region versus region comparisons of gene expression within each Cell Subtype (for regions that had corresponding cell subtypes) and within either HX or NX. Statistically significance (“sig” column in all tables) was defined as |log_2_(FC) | > 0.25 and FDR < 0.05. Download Table 5-1, XLS file.

We expanded our findings from the differential gene expression analysis by focusing on understanding cell–cell signaling after neonatal HX. Neighboring cells communicate via secreted molecules (ligands) that can act on receptors in nearby cells. These ligand–receptor (LR) networks are fundamental signaling nodes that regulate circuit function and injury response. To understand how local cell–cell communication networks change in the context of neonatal brain injury, we performed a spatially informed LR analysis using CellChat v2 (see Materials and Methods). We grouped predicted LR pairs based on whether signaling occurred within the same cell type (e.g., OPC to OPC; intralineage) or between different cell types (e.g., microglia to OPC; interlineage). After exposure to neonatal HX, we identified region-specific cellular programs governing regenerative oligodendrogenesis and neurogenesis.

**Figure 6.**
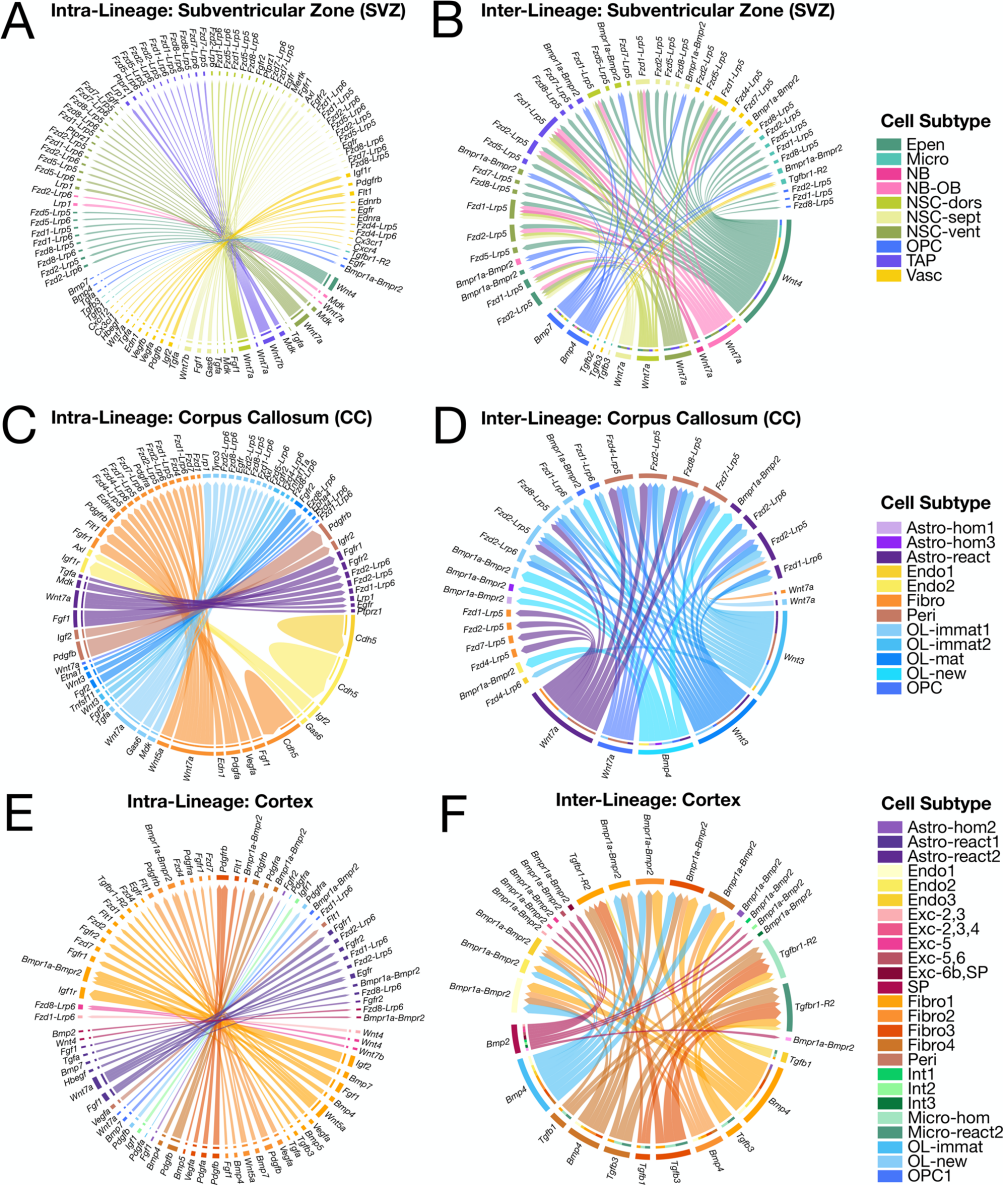
Cell subtypes exhibit discrete cell–cell signaling networks in the brains of mice exposed to neonatal hypoxia. Chord diagrams of predicted intralineage cell–cell communication pathways that are upregulated in the (***A***) SVZ, (***B***) corpus callosum, and (***C***) cortex of HX versus NX mice. Chord diagrams of predicted interlineage cell–cell communication pathways that are upregulated in the (***D***) SVZ, (***E***) corpus callosum, and (***F***) cortex of HX versus NX mice. Cell Subtype identity of the ligand is indicated on the outermost edge of the diagram, while the Cell Subtype identity of the receptor is indicated by the internal ring. Encoding gene symbols are used to represent predicted interactions of their protein products. Colored arrows indicate the specific ligand–receptor (LR) pairs and are colored according to the outgoing ligand signal. For all chord diagrams, only significant ligand and receptor interactions are plotted (*p* < 0.05). Full results are shown in Extended Data Table 6-1.

In the SVZ, we found that HX induced upregulation of *Egfr*, *Lmnb1*, and the proliferation marker *Mki67* within most SVZ cell types, including the neural progenitors NSCs, OPCs, and TAPs, suggestive of NSC activation (Fig. 5*G* and Extended Data Table 5-1; Koufi et al., 2023). In response to HX, neural progenitors also upregulated genes involved in OL differentiation such as *Qk* and *Ptprz1*, and neurogenic genes including *Dcx*, *Gria2*, and *Stmn1*, suggestive of increased neurogenic and gliogenic priming (Extended Data Table 5-1). We observed dramatic changes in BMP, WNT, and Notch signaling in the SVZ, consistent with their role in regulating stem and progenitor cell maintenance, proliferation, differentiation, and fate decisions (Figs. 5*G*, 6*A,B*, Extended Data Tables 5-1, 6-1). Specifically, there was a significant increase in *Bmp7* expression in OPCs (Fig. 5*G*) and an increase in BMP7 and BMP4 signaling from OPCs to OPCs, NSCs, TAPs, microglia, and vascular cells (Fig. 6*A,B*). In addition, the WNT ligand *Wnt7a*, the receptor *Lrp6*, the effector *Ctnnb1*, and target gene *Ccnd1* all demonstrated increased expression in most SVZ cells, including NSCs, after HX (Fig. 5*G* and Extended Data Table 5-1) which translated to a widespread increase in WNT signaling (Fig. 6*B* and Extended Data Table 6-1). In the HX SVZ, *Notch1* and *Notch2* receptors were upregulated in most cell types, while the ligand genes *Dll1* or *Dll4* were upregulated in vascular cells, TAPs, and immune cells (Fig. 5*G* and Extended Data Table 5-1) which translated to an increase in Notch signaling between these cell types (Extended Data Table 6-1). Interestingly, we found increased transforming growth factor β-2 and 3 (TGFβ-2/3) signaling between the vasculature and microglia in the SVZ after HX (Fig. 6*B* and Extended Data Table 6-1). TGFβ is a critical component of the microglial proneurogenic response (Battista et al., 2006; Makwana et al., 2007; De Lucia et al., 2016).

10.1523/ENEURO.0224-24.2024.t6-1 Table 6-1 **Ligand-receptor analysis**. This table is available at available at https://figshare.com/s/f096946cf6543f034528. Ligand-receptor analysis of intracellular and intercellular communication comparing HX versus NX differences within each Cell Type and Cell Subtype in the cortex, SVZ, and corpus callosum. Statistical significance (“sig” column in all tables) was defined as having p-value < 0.05. Download Table 6-1, XLS file.

In the corpus callosum, we identified several signaling nodes that promote white matter recovery after neonatal HX, including upregulation of MAG to MAG and TGF-α to epidermal growth factor receptor (EGFR) signaling between Cell Subtypes of OL-lineage cells (Extended Data Table 6-1). We observed increased WNT signaling from astrocytes and OLs, increased IGF2/IGF1R signaling from vascular cells to astrocytes and OLs, and increased CNTN1-Notch signaling between OLs, astrocytes, and vascular cells (Extended Data Table 6-1). Changes in Notch signaling were a commonly identified theme in the corpus callosum: *Notch1* and *Notch2* were upregulated in astrocytes and OL-lineage cells and the ligand *Dll1* was upregulated in OL-lineage cells (Fig. 5*G* and Extended Data Table 5-1). Moreover, we found increased GAS6 signaling through its receptors (TAM receptors, encoded by *Tyro3*, *Axl*, and *Mer*) between microglia and astrocytes, vascular cells, and OL-lineage cells (Extended Data Table 6-1). Following demyelinating injury, GAS6-TAM signaling promotes remyelination and glial cell development, including suppression of a deleterious (antiregenerative) microglia response (Binder et al., 2011, 2008). Notably, we found evidence of neurovascular adaptation after HX, including upregulation of genes involved in cell migration and vascular permeability (*Fstl1* and *Itgb1*; Fujioka et al., 2017) in vascular cells in the corpus callosum, which may promote immune cell infiltration and/or guide the migration of new neurons and glial cells (Fig. 5*G* and Extended Data Table 5-1). We observed an increase in inflammatory and migratory signaling from corpus callosum fibroblasts, including upregulation of Interleukin 34 (IL-34) to colony-stimulating factor 1 receptor (CSF1R) and ephrin A1 (EFNA1) to ephrin type A receptor 7 (EphA7) ligand–receptor pairs, both of which may support glial scar formation (Fig. 6*C,D* and Extended Data Table 6-1; Gerber et al., 2018; Bellver-Landete et al., 2019; Zhou et al., 2023).

In the cortex, LR analysis was notable for a broad, multicellular increase in several signaling pathways previously implicated in brain circuit wiring and the response to injury. Endothelin-1 (EDN1)/endothelin receptor type B (EDNRB) signaling was increased between endothelial cells, fibroblasts, astrocytes, OLs, microglia, and astrocytes (Extended Data Table 6-1). Endothelin regulates myelination capacity in OLs, as endothelin is upregulated after demyelination and directly inhibits OPC differentiation via astrocytes (Hammond et al., 2015). Additionally, we found that fractalkine (encoded by *Cx3cl1*)-CX3CR1 signaling, a regulator of gliogenesis, was increased between several non-neuronal cell populations, including OLs, OPCs, fibroblasts, and astrocytes, to reactive microglia (Extended Data Table 6-1). A regulator of fractalkine expression and remyelination, TGFβ1 signaling, was also enhanced following HX between endothelial cells, fibroblasts, and microglia. Canonical WNT signaling is important for TGFβ-mediated fibrosis (Akhmetshina et al., 2012), and we identified increased WNT signaling in deep layer cortical excitatory neurons (WNT4 with FZD4), as well as between vascular cells, deep layer excitatory neurons, and astrocytes (WNT7A with FZD4). We observed an HX-induced increase in cytokine signaling (including IGF2-IGF1R, IGF2-IG2R, fractalkine-CX3CR1, TGFB1-TGFBR1, and TGFB3-TGFBR1 ligand–receptor pairs) largely sourced by fibroblasts and other vascular cells and targeting vascular cells and microglia (Fig. 6*E,F* and Extended Data Table 6-1). Thus, vascular cells may represent an important source of inflammatory signaling leading to increased microglial activation after neonatal HX. Additionally, several homophilic (CDH5-CDH5, ESAM-ESAM, PECAM1-PECAM1) and heterophilic (FGF1/2-FGFR1/2R, BMP4/7-BMPR1A, VEGFA-FLT1) endothelial interactions involved in regulating vascular development and blood–brain barrier permeability integrity were dysregulated in all three regions of the brain (Fig. 6*A–F* and Extended Data Table 6-1; Argaw et al., 2012; W. Li et al., 2018; Klimaschewski and Claus, 2021; Knox et al., 2022). In addition, in the cortex, we found that HX induced an upregulation of *Sst* expression in interneurons and an upregulation of *Sstr2* expression in both interneurons and excitatory neurons (deep and superficial layers). Furthermore, LR analysis revealed increased *SST-SSTR2* signaling from interneurons to other neurons following HX (Extended Data Table 6-1), all of which could impact excitatory–inhibitory neuron balance.

Ephrin communication was commonly altered across the cortex, SVZ, and corpus callosum. Cortical excitatory neurons and interneurons in the HX brain showed an upregulation of receptors *Epha4*, *Epha6*, and *Epha7* and ligands *Efnb2* and *Efnb3* (Fig. 5*G* and Extended Data Table 5-1). EFNA1 signaling by cortical fibroblasts to OL-lineage cells and astrocytes was significantly upregulated post-HX (Extended Data Table 6-1). In the SVZ, there was widespread upregulation of ephrin genes; for example, *Epha4* and *Efnb2* were upregulated in all cell types following HX (Extended Data Table 5-1). Furthermore, all SVZ cells participated in ephrin signaling as sources and targets following HX. The OL lineage in the corpus callosum also upregulated ephrin genes and showed increased ephrin signaling targeting all major Cell Types (Extended Data Table 6-1). EFNA1 interacts with EphA4 to inhibit OL process extension via ephexin1-RhoA-Rock-myosin 2 (Harboe et al., 2018). These HX-induced ephrin interactions may play an important role in guiding the migration and process extension of injury-responsive glial cells or new neurons.

Another important source of cell guidance signaling is the extracellular matrix. In particular, chondroitin sulfate proteoglycans (CSPGs) are extracellular matrix components known to regulate cell migration, synapse formation and maturation, and inflammation in the developing brain (Siebert et al., 2014; Dyck and Karimi-Abdolrezaee, 2015). However, the upregulation of CSPGs following brain injury has been shown to block the migration of neuroblasts and inhibit regeneration (Siebert et al., 2014; Luo et al., 2022). We found that OL-lineage cells and astrocytes in the cortex and corpus callosum, cortical excitatory neurons, cortical interneurons, and all SVZ cell types showed an upregulation of some CSPG genes such as *Vcan*, *Ncan*, *Bcan*, and *Tnr* (Fig. 5*G* and Extended Data Table 5-1). To examine whether the increase in CSPG gene expression correlated with an increase in protein levels, we performed immunofluorescence staining for NCAN in the P21 brain. We found that the average positive fluorescence intensity of NCAN staining over the area was significantly higher across the HX cortex compared with NX (Fig. 5*H,I*). Furthermore, the significant increase in NCAN fluorescence intensity was true across the superficial, middle, and deep cortical layers (Fig. 5*J*). These results indicate an increase in neural guidance cues in the extracellular matrix that could affect the appropriate migration and integration of new cells in the HX brain.

### Neonatal HX is associated with changes in region-specific cell states

**Figure 7.**
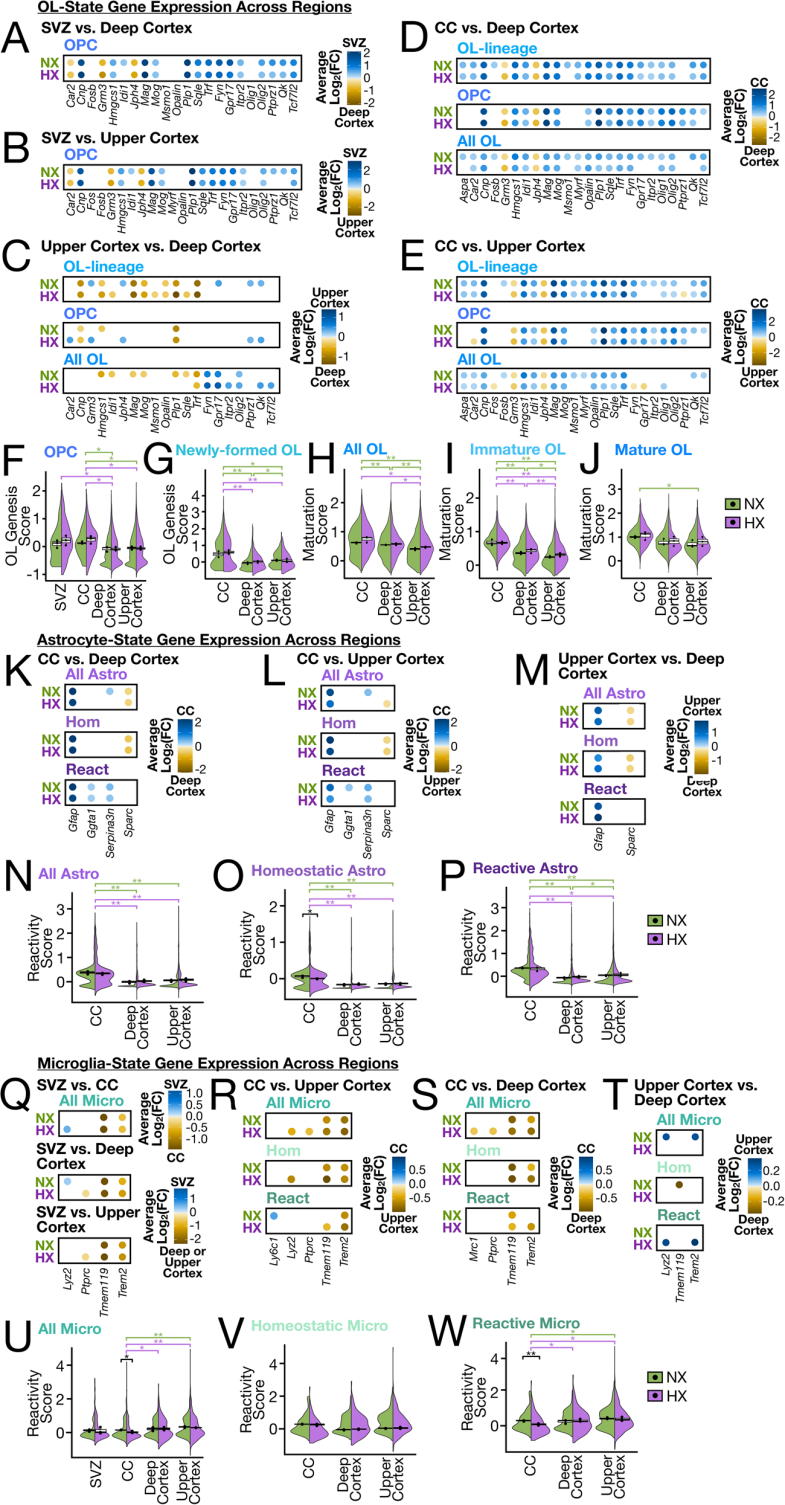
Cell state changes of OL-lineage cells, astrocytes, and microglia across anatomical regions and following neonatal hypoxia exposure. Dotplots displaying differential gene expression results of OL-state (OL genesis and OL maturation) genes comparing all OL-lineage cells (Cell Type) and Cell Subtypes within NX and HX between the (***A***) SVZ and deep cortex, (***B***) SVZ and upper cortex, (***C***) upper and deep cortex, (***D***) corpus callosum (CC in all panels) and deep cortex, and (***E***) corpus callosum and upper cortex. Violin boxplots showing OL genesis state score in (***F***) newly-formed OLs and (***G***) OPCs, and OL maturation score of (***H***) all OLs, (***I***) immature OLs, and (***J***) mature OLs, split and colored by condition across the SVZ, corpus callosum, and cortex. Dotplots displaying differential gene expression results of astrocyte reactivity genes comparing all astrocyte Cell Types and Cell Subtypes within NX and HX between (***K***) corpus callosum and deep cortex, (***L***) corpus callosum and upper cortex, and (***M***) upper and deep cortex. Violin boxplots showing astrocyte reactivity score of (***N***) all astrocytes, (***O***) homeostatic astrocytes, and (***P***) reactive astrocytes, split and colored by condition, across the SVZ, corpus callosum, and cortex dotplots displaying differential gene expression results of microglia reactivity genes comparing all microglia Cell Types and Cell Subtypes within NX and HX between the (***Q***) SVZ and corpus callosum, SVZ and deep cortex, SVZ and upper cortex, (***R***) corpus callosum and deep cortex, (***S***) corpus callosum and upper cortex, and (***T***) upper cortex and deep cortex. Violin boxplots showing microglia reactivity score of (***U***) all microglia, (***V***) homeostatic microglia, and (***W***) reactive microglia, split and colored by condition, across the SVZ, corpus callosum, and cortex. For all dotplots, only significant genes are shown (FDR < 0.05), and the dot color represents average log_2_(FC) as per the associated legend. Results for all dotplots are also shown in Extended Data Table 5-1. For all violin boxplots, the width of the violin plot represents the proportion of cells expressing that value of the score. Each dot represents the average score for each replicate, the bold central line of the boxplot represents the median score for the condition, and the limits of the box represent the interquartile range. Significance is determined using the two-tailed Student's *t* test. *p < 0.05; **p < 0.01. Asterisks denoting *p*-value are colored according to the comparison they refer to (i.e., turquoise denotes comparisons of different regions within NX, purple denotes comparisons of different regions with HX, and black denotes comparisons between HX and NX within the same region).

Finally, we asked whether microglia, astrocyte, and OL-lineage cell state is dictated by spatial context by comparing gene expression patterns between the SVZ, corpus callosum, and cortex. Differential gene expression analysis revealed notable differences in cell state-associated gene expression across different anatomical regions in the NX brain. OL-lineage cells in different regions showed different levels of gene expression for OL differentiation- and maturation-related genes. OPCs in the SVZ (Fig. 7*A,B*) and corpus callosum (Fig. 7*D,E*) had higher levels of many oligodendrogenesis genes including *Olig2*, *Gpr17*, *Itpr2*, and *Fyn* compared with both superficial and deep layer cortical OPCs. Following HX, these genes were upregulated in SVZ OPCs while only some (i.e., *Gpr17*) were upregulated in corpus callosum OPCs (Fig. 5*K* and Extended Data Table 5-1). To characterize the overall differentiation and maturation state of OL-lineage cells, we used several established genes (Marques et al., 2016; Floriddia et al., 2020; Takeuchi et al., 2020) to calculate scores for OL differentiation priming (“OL genesis”) and OL maturation. We found that OPCs in the SVZ and corpus callosum had a higher OL genesis score than cortical OPCs (Fig. 7*F*). Similarly, newly formed OLs in the corpus callosum had a higher OL genesis score than in the cortex (Fig. 7*G*). Following HX, there was a trending but statistically not significant increase in the OL genesis scores among OPCs in the SVZ and corpus callosum, as well as in newly formed OLs in the corpus callosum (Fig. 7*F,G*). The OL maturation score was higher in the corpus callosum than in the superficial and deep cortical layers for OL-lineage cells altogether (Fig. 7*H*) and specifically in immature (Fig. 7*I*) and mature OLs (Fig. 7*J*). This score also showed a trending but not significant increase in upper cortical OLs following HX (Fig. 7*H–J*). Overall, these results suggest regional differences in the maturation dynamics between corpus callosum and cortical OLs. However, the weak effect of HX on oligodendrogenic potential and maturation suggests that myelination impairments associated with DWMI may have less to do with OL dysfunction and more with the influence of their cellular environment.

In the both NX and HX brains, all astrocytes in the corpus callosum had significantly higher expression of *Gfap* than astrocytes in the cortex, with Astro-react also exhibiting higher levels of reactive markers *Ggta1* and *Serpina3n* in the corpus callosum (Fig. 7*K–M*). Following HX, all astrocytes in the cortex, but not the corpus callosum upregulated *Gfap* (Fig. 5*L*). We then calculated astrocyte reactivity scores from established reactive astrocyte genes (Zamanian et al., 2012; Liddelow et al., 2017; Matusova et al., 2023) and found that astrocytes in the NX corpus callosum exhibited a significantly higher state of reactivity compared with those in the cortex. This heightened reactivity was not altered by HX treatment. Within the cortex, astrocytes in the superficial layers showed a slightly elevated reactivity score over those in the deep layers (Fig. 7*N–P*). Astro-hom, on the other hand, had higher *Sparc* expression in the cortex than in the corpus callosum, with deep layer astrocytes expressing more *Sparc* than superficial ones, a trend maintained post-HX (Fig. 7*K–M*). Since *Sparc* encodes a secreted protein that negatively regulates synaptogenesis (Kucukdereli et al., 2011), this emphasizes the specialized function of cortical astrocytes in synapse formation and neural homeostasis.

Differential gene expression analysis revealed that microglia in both the NX and HX SVZ and corpus callosum exhibited a significantly lower expression of both *Tmem119* and *Trem2* compared with microglia in the superficial cortex and deep cortex (Fig. 7*Q–T*). SVZ microglia showed the same trend of lower expression of both *Tmem119* and *Trem2* when compared with the corpus callosum (Fig. 7*Q*). This trend is unexpected because it is widely acknowledged that *Tmem119* has higher expression in homeostatic microglia while *Trem2* is higher in reactive microglia (Boche and Gordon, 2022). However, recent work showing that *Tmem119* gene expression levels and spatial distribution in the brain are incongruent with protein levels following traumatic brain injury challenges the use of transcriptomic *Tmem119* as a marker for homeostatic microglia (Mercurio et al., 2022). Therefore, to better characterize the reactivity state of microglia across different regions, we calculated reactivity scores using multiple genes shown previously to be specific to or enriched in reactive microglia at the transcriptomic level (see Materials and Methods; Wahane et al., 2021; Paolicelli et al., 2022). We observed a lower reactivity score in corpus callosum microglia, specifically Micro-react, compared with superficial cortex microglia (Fig. 7*U–W*). HX exposure reduced the reactivity score of corpus callosum microglia significantly, pushing microglia reactivity in the corpus callosum to be significantly lower than both upper and deep cortex microglia. In addition, only microglia in the SVZ exhibited a significant change in state-related genes following HX exposure, including an upregulation of homeostatic microglia-associated *Tmem119* and reactive marker *Lgals3* (Fig. 5*M*).

## Discussion

Advances in the care of very low birth weight preterm infants have markedly improved survival. However, despite these advances, survivors of preterm birth continue to have a high propensity for developmental delay and disability. Chronic HX exposure resulting from an underdeveloped respiratory system leads to cerebral WMI, a major cause of neurodevelopmental disorders in preterm infants, including cerebral palsy. However, the underlying cellular mechanisms that govern the brain's response to HX and recovery from WMI remain poorly understood. Here, we employed high-resolution spatial transcriptomics as a discovery platform to map cell type- and region-specific transcriptional signatures during recovery from neonatal HX. At baseline (NX condition), we identified several brain region-specific cell states, including spatially restricted populations of glia and vascular cells that play a key role in modulating the signaling environment and may regulate developmental processes such as neurogenesis and gliogenesis, cell migration, synapse formation, and inflammation. We found that during the reparative phase following neonatal HX exposure, there were changes in regional cell type composition, neurogenic and gliogenic transcriptional programs, and local cellular architecture, which are likely the result of extensive changes in regional signaling networks. Thus, our results support a model of niche cell states and signaling networks that are region- and stimulus-dependent.

First, we demonstrate that at baseline, OPCs in the neurogenic niche and white matter have a transcriptomic profile that is more proliferative and primed for differentiation than in the cortex. In response to HX, OPCs within the neurogenic niche further upregulate proliferation-associated genes and genes encoding ligands that modulate OL formation and myelination. We found that OPCs upregulated BMP ligand genes such as *Bmp4* which, in the context of demyelinating injury, has been shown to be produced by injury-activated OPCs and inhibit the differentiation of OPCs to OLs (Ulanska-Poutanen et al., 2018). We also observed upregulation of *Cx3cl1*, which encodes fractalkine, from several glial cell types including OL-lineage cells to reactive microglia. Fractalkine has an established pro-regenerative role in remyelination, particularly via OPCs and microglia (Ridderstad Wollberg et al., 2014; de Almeida et al., 2023). Interestingly, previous work has shown that environmental enrichment both enhances recovery from neonatal diffuse WMI and increases fractalkine levels. Importantly, TGFβ is known to regulate fractalkine expression, and we identified increased TGFβ1 signaling among glial and vascular cell types, which likely acts as an upstream regulator of tissue remodeling after HX. While OPCs in the white matter became more abundant, newly formed OLs became less abundant, suggesting differentiation arrest, specifically in the transition from OPCs to newly formed OLs.

Furthermore, we found that developing and mature OLs from the cortex and corpus callosum exhibited different cell states and responded differently to HX. At baseline, white matter OLs overall were more mature than cortical OLs (exhibiting higher OL maturation scores), and these levels did not change significantly following HX. Analysis of interlineage cell–cell communication revealed alterations in GAS6-TAM, EDN1-EDNRB, and fractalkine-CX3CR1 networks in the cortex that may play a direct role in promoting OPC differentiation and altering OL myelination capacity in the context of neonatal HX. In addition, we identify significant changes in the spatial organization of homeostatic astrocytes. Previous studies have shown that astrocyte–OL interactions are crucial in facilitating OPC differentiation into mature myelin-forming OL via a process orchestrated through the Nrf2 pathway (Molina-Gonzalez et al., 2023).

Importantly, we identified widespread changes in extracellular matrix components, including CSPGs, after HX. CSPGs contribute to the formation of perineuronal nets during postnatal development and play a role in the closure of the critical window of plasticity (Hensch, 2005; Fawcett et al., 2019). We observed that in most cell types in the cortex and corpus callosum, neonatal HX was associated with increased expression of genes important for cell migration and projection guidance including ephrins and CSPGs. These signals may play an important role in coordinating the migration of injury-responsive cells such as immune cells and astrocytes, as well as migrating upper layer excitatory neurons. Interestingly, we did find that the abundance and local organization of upper layer excitatory neurons was disrupted by HX. However, it is unclear whether the upregulation of guidance cues such as CSPGs would help or hinder appropriate cell migration. Depletion of CSPGs in the adult brain by enzymatic degradation or genetic inhibition has been shown to promote plasticity (Lensjø et al., 2017; Reichelt et al., 2019; Willis et al., 2022). CSPGs can also act as potent inhibitors of myelination, axon guidance and regeneration, and other injury repair processes. Further work is needed to determine the clinical relevance of CSPGs in myelin recovery after neonatal WMI.

While most studies on neonatal WMI have focused on OL-lineage cells, our study underscores the complex and region-specific multicellular signaling that may support gliogenesis but also ultimately lead to impaired functional connectivity. Specifically, we found that endothelial cells, fibroblasts, and pericytes were major sources of signaling molecules and that their phenotype changed dramatically following HX in a region-specific manner. Our findings imply that cross-talk signaling from vasculature-associated cells may be crucial for activating angiogenesis and forming adhesive interactions that maintain blood–brain barrier integrity and modulate permeability after HX exposure. Additionally, vascular cells may represent an important source of inflammatory ligands that promote microglial reactivity after neonatal HX. We also found that corpus callosum microglia may be less reactive than cortical microglia under NX and HX conditions, which contrasts with previous research in adult rodents and humans, suggesting that white matter microglia are more reactive to injury than gray matter microglia (Batchelor et al., 2008; van der Poel et al., 2019). As these earlier studies primarily involved adult and aged rodents, our findings reflect a different, developmental microglia state. Specifically, the heightened cortical microglial reactivity observed in our study may relate to their role in synapse refinement during brain development following HX exposure, although this requires further investigation.

An important strength of the present study is the use of MERFISH, which enables single-cell resolution spatial transcriptomics. Previous work on neonatal HX has focused largely on the corpus callosum, but paracrine and noncell autonomous mechanisms of recovery after neonatal brain injury are poorly understood. The present analysis spans most major anatomical brain regions and highlights regional variability in the vulnerability and response to injury. Nonetheless, there are several limitations to the present study. Firstly, the mouse model of chronic neonatal HX does not introduce inflammatory stimuli, which are often an important component of the encephalopathy of prematurity. Additionally, the sublethal chronic HX model employed does not fully replicate the intermittent hypoxic events experienced by many premature infants. Notably, the pattern of WMI in preterm infants has become less severe in the last two decades, and thus, cerebral dysmaturation may also be contributing to neurodevelopmental delays and impairments (Schneider and Miller, 2019). From a methodologic perspective, MERFISH requires segmentation of cell boundaries to assign transcripts to individual cells. We employed a machine learning algorithm and extensive manual curation, including stringent doublet elimination, to mitigate limitations of cell boundary identification. However, precise cell boundary identification remains an ongoing challenge for in situ spatial transcriptomics, and mis-segmentation may introduce false cell type assignment of gene expression. While our analysis was limited to a single time point, the objective of the study was to identify pathways critical for recovery and repair after neonatal HX. Finally, commercial MERFISH methods are presently limited to a finite number of genes, so additional changes at the level of the whole transcriptome were not captured.

Despite these limitations, this work presents an extensive spatially informed landscape of gene expression changes in an established mouse model of neonatal brain injury. We developed and employed a new approach to mapping physical cellular interactions using imaging-based spatial transcriptomics, which can be extended to other developmental and disease contexts. Our whole-brain analysis is a foundational resource for developmental WMI and for regenerative cellular communication.

## Synthesis

Reviewing Editor: Julie Bakker, University of Liege Decisions are customarily a result of the Reviewing Editor and the peer reviewers coming together and discussing their recommendations until a consensus is reached. When revisions are invited, a fact-based synthesis statement explaining their decision and outlining what is needed to prepare a revision will be listed below. The following reviewer(s) agreed to reveal their identity: NONE.

Before the ms could be accepted, the authors should make some necessary changes as outlined by the reviewer:

The authors modified the first section of the manuscript by removing unnecessary gene names, making it clearer and more straightforward to understand. Additionally, the figures were updated accordingly, effectively presenting complex ideas in an accessible manner and enhancing the clarity and logical flow of the manuscript.

However, there are still a few minor issues to resolve:

• The authors should choose and consistently use either the term 'perinatal hypoxia' or 'neonatal hypoxia' throughout the manuscript; both cannot be used interchangeably. From the perspective of the mouse model, the correct term is 'neonatal,' which I suggest using.

• Pages 16 and 17 and others: There is no Figure 3C (only C' and C"). Please ensure consistency throughout the text and Figure.

• Page 20, line 489: Instead of phrase 'all other regions' authors should specify which regions are being referred to.

• Page 20, line 509: An increase in Endo-1 cells in Figure 2M does not appear to be of 82% magnitude.

• In Figure 2L, M, and others, where the authors compare hypoxia and normoxia, the color choices make the differences difficult to discern, especially for individuals with visual impairments. I suggest using a more contrasting color scheme, preferably color-blind-friendly, to more clearly express the differences between the conditions.

• Figure captions: Authors use capital letters for two purposes: to refer to figure panels or brain tissue areas, thus creating confusion for a reader, even though the former are in boldface (however inconsistently! - lines 125, 126, 128, etc). I suggest using lowercase for brain tissue area references vs capital boldface for figure panel.

• Figure 2 caption: OPC acronym remains uncorrected - it should be replaced with OPC1, and OPC2 should be added to correctly reflect figure content.

Once these final adjustments are made, I am confident that the manuscript will not only meet the journal's rigorous standards for acceptance but will also present a comprehensive and polished data on the cell type classification and special distribution in the mouse brain in the physiological and hypoxic conditions that contributes valuable insights to the field of brain injury induced by hypoxia. The revisions will enhance the clarity and impact of the findings, positioning the manuscript strongly for publication and increasing its potential to resonate with a wide academic audience.
