# Supplementary figures and images for: Neonatal Brain Injury Triggers Niche-Specific Changes to Cellular Biogeography
Source: eNeuro. 2024 Dec 19;11(12):ENEURO.0224-24.2024. doi: 10.1523/ENEURO.0224-24.2024 (PMC11680506; doi:10.1523/ENEURO.0224-24.2024)

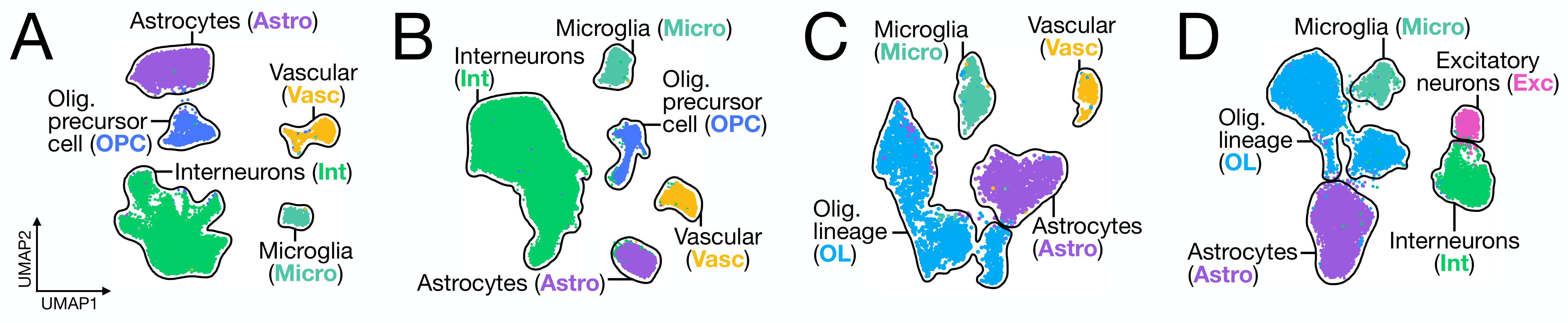

Supplement: Figure 2-1 — Cell Type identification across the P21 brain. UMAP visualization of cell types identified in the (A) septum, (B) caudate putamen (CP), (C) anterior commissure (ACo), and (D) septal white matter tracts (SWM), where each dot represents a single cell. UMAP plots are generated from combined replicates across NX and HX conditions. Each cluster is colored by Cell Type. Download Figure 2-1, TIF file. [file eneuro-11-ENEURO.0224-24.2024-s001.tif]

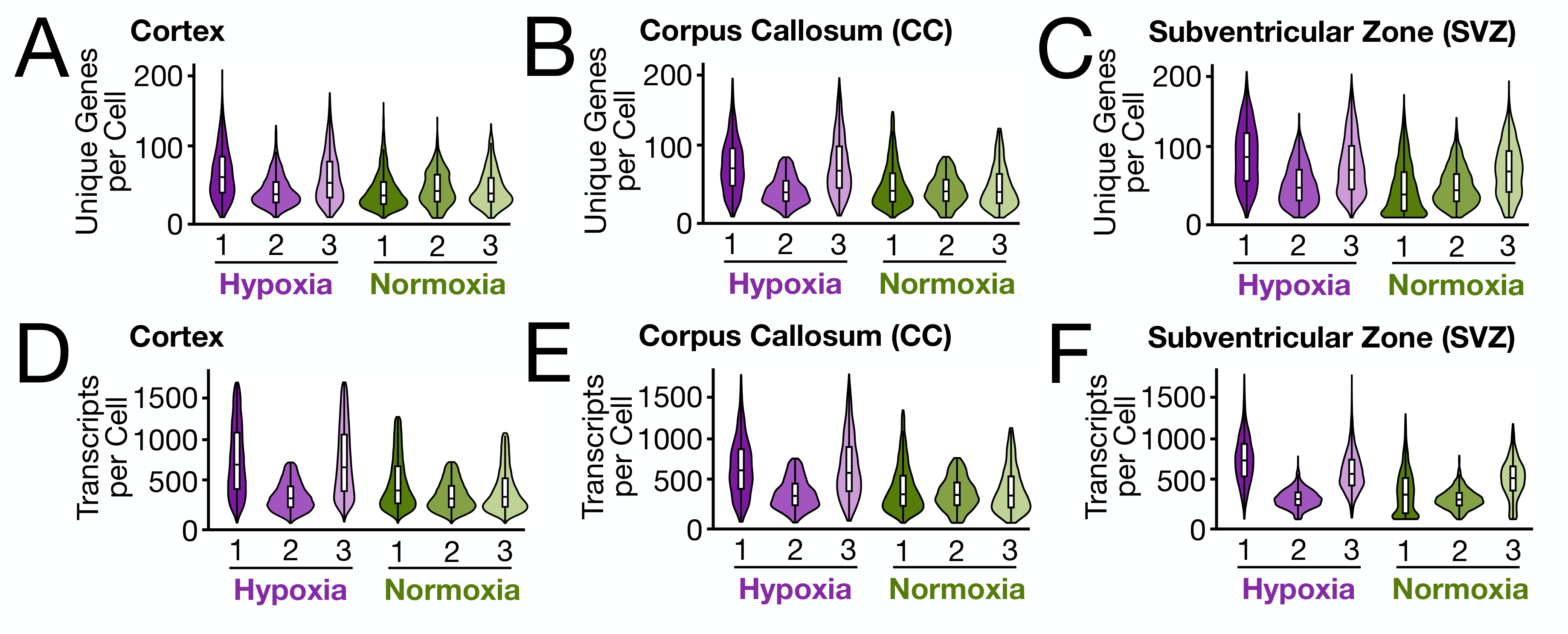

Supplement: Figure 2-2 — Quality control metrics of the MERFISH data. Violin plots showing select quality control parameters used to profile the data, including the number of unique genes per cell in the (D) cortex, (E) corpus callosum, and (F) SVZ, and the number of transcripts per cell in the (G) cortex, (H) corpus callosum, and (I) SVZ. Download Figure 2-2, TIF file. [file eneuro-11-ENEURO.0224-24.2024-s002.tif]

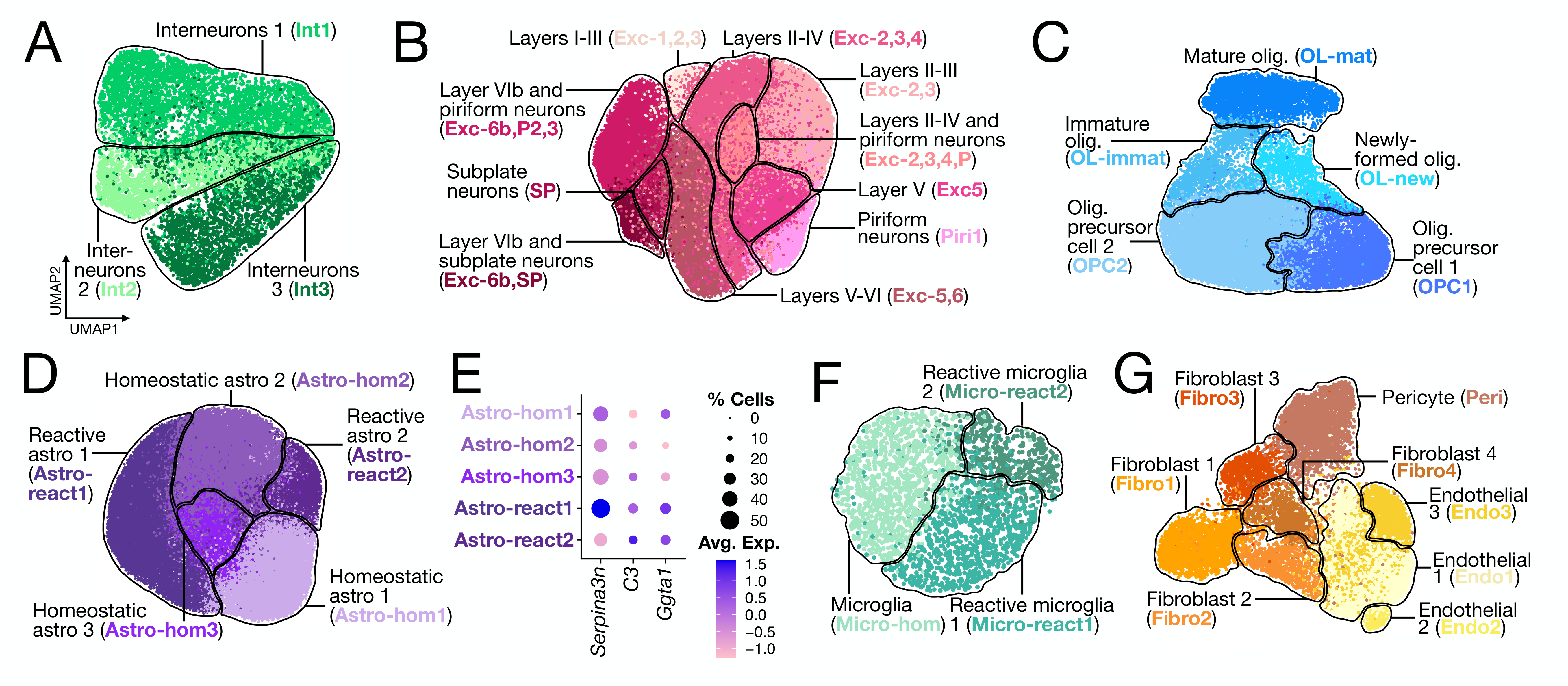

Supplement: Figure 2-3 — Cell Subtype identification in the cortex of the P21 brain. (A-D, F-G) UMAP visualization of cortical (A) interneurons and (B) excitatory neurons, (C) OL-lineage cells, (D) astrocytes, (F) microglia, and (G) vascular cells. UMAP plots are generated from combined replicates across NX and HX conditions. Each dot represents a single cell, and clusters of cells are colored by Cell Subtype as previously defined. (E) Dotplot displaying average expression level of reactive astrocyte genes in all astrocyte Cell Subtypes in (D). The dotplot is intended to clarify the data shown in Figure 2A where the range of gene expression in the violin plots are too wide to visually depict the average expression level of these genes. Dot size indicates the percentage of cells in the group where the gene is detected, and color indicates average expression level of the gene within the group. Download Figure 2-3, TIF file. [file eneuro-11-ENEURO.0224-24.2024-s003.tif]

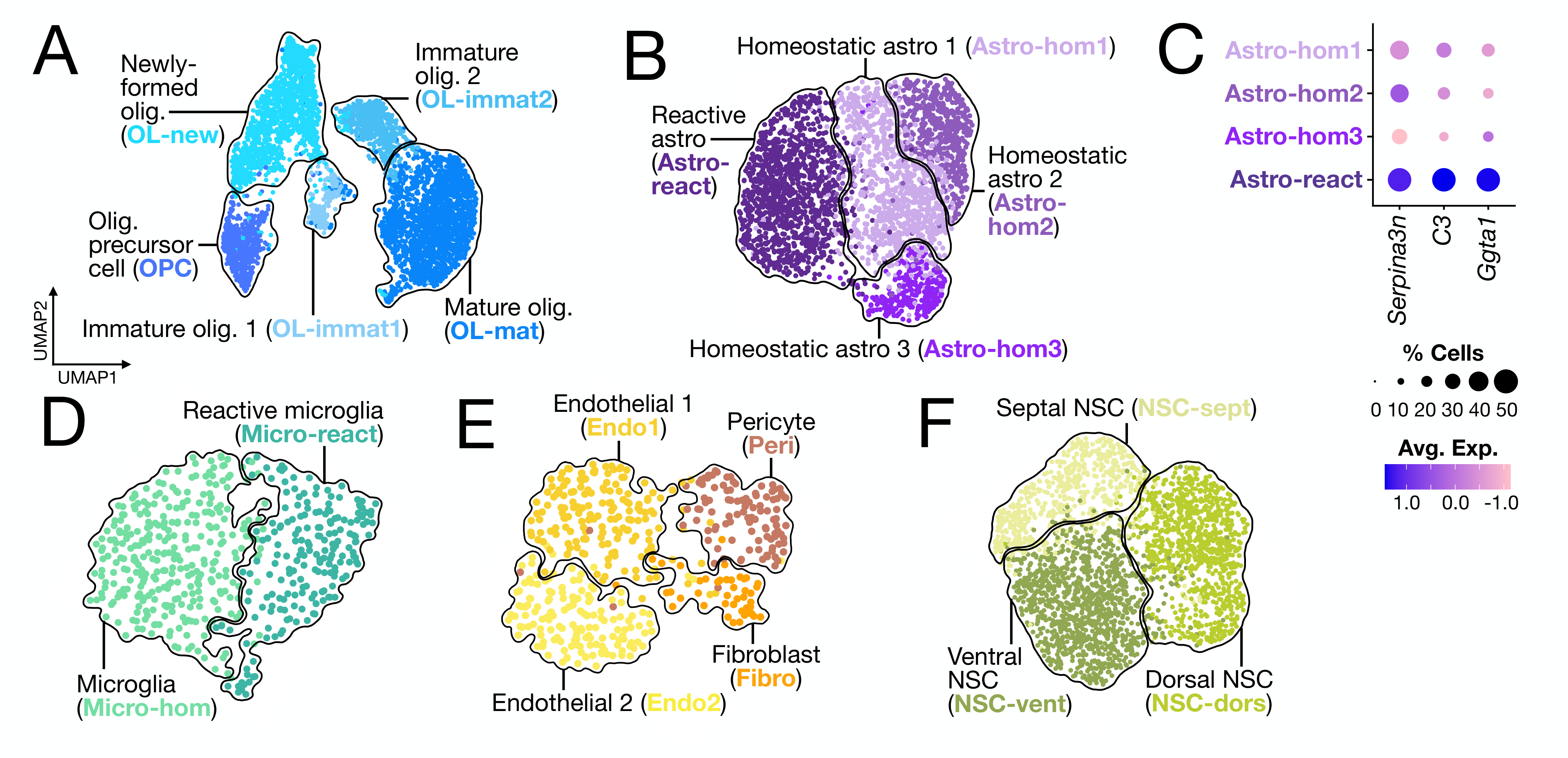

Supplement: Figure 3-1 — Cell Subtype identification across the cortex, SVZ, and corpus callosum of the P21 brain. (A-B, D-F) UMAP visualization of corpus callosum (A) OL-lineage cells, (B) astrocytes, (D) microglia, (E) vascular cells; and SVZ (F) neural stem cells (NSC). UMAP plots are generated from combined replicates across NX and HX conditions. Each dot represents a single cell, and clusters of cells are colored by Cell Subtype as previously defined. (C) Dotplot displaying average expression level of reactive astrocyte genes in all astrocyte Cell Subtypes in (B). This dotplot is intended to clarify the data shown in Figure 3B where the range of gene expression in the violin plots are too wide to visually depict the average expression level of these genes. Dot size indicates the percentage of cells in the group where the gene is detected, and color indicates average expression level of the gene within the group. Download Figure 3-1, TIF file. [file eneuro-11-ENEURO.0224-24.2024-s004.tif]

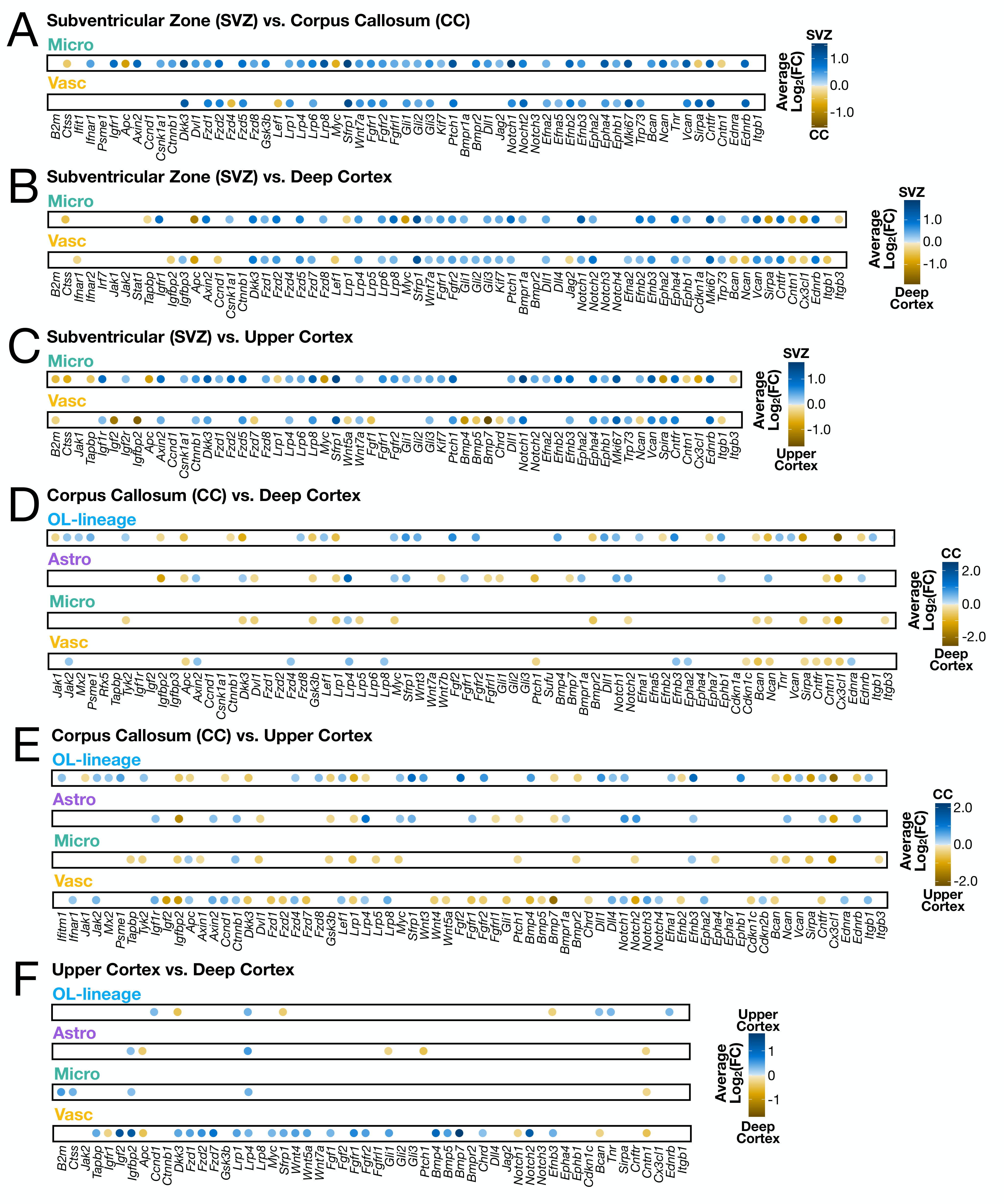

Supplement: Figure 5-1 — Comparison of signaling-related gene expression between anatomical regions in mice exposed to normoxic conditions. Dotplots displaying differential gene expression results comparing Cell Types between SVZ, corpus callosum, deep cortex, and upper cortex within NX mice. Only significant genes (FDR < 0.05) are shown. The color of each dot represents the average log2(FC) as per each associated legend. Comparisons shown are (A) SVZ versus corpus callosum, (B) SVZ and deep cortex, (C) SVZ versus upper cortex, (D) corpus callosum versus deep cortex, (E) corpus callosum versus upper cortex, and (F) deep cortex versus upper cortex. Full results, including genes not depicted in these graphs, are shown in Table 5-1. Download Figure 5-1, TIF file. [file eneuro-11-ENEURO.0224-24.2024-s005.tif]
